# Transient Adaptation of *Toxoplasma gondii* to Exposure by Thiosemicarbazone Drugs That Target Ribosomal Proteins Is Associated with the Upregulated Expression of Tachyzoite Transmembrane Proteins and Transporters

**DOI:** 10.3390/ijms25169067

**Published:** 2024-08-21

**Authors:** Manuela Semeraro, Ghalia Boubaker, Mirco Scaccaglia, Joachim Müller, Anitha Vigneswaran, Kai Pascal Alexander Hänggeli, Yosra Amdouni, Laura Helen Kramer, Alice Vismarra, Marco Genchi, Giorgio Pelosi, Franco Bisceglie, Manfred Heller, Anne-Christine Uldry, Sophie Braga-Lagache, Andrew Hemphill

**Affiliations:** 1 Department of Veterinary Medicine Sciences, University of Parma, Strada del Taglio 10, 43126 Parma, Italy; manuela.semeraro@unipr.it (M.S.); laurahelen.kramer@unipr.it (L.H.K.); alice.vismarra@unipr.it (A.V.); marco.genchi@unipr.it (M.G.); 2 Institute of Parasitology, Department of Infectious Diseases and Pathobiology, Vetsuisse Faculty, University of Bern, Länggass-Strasse 122, 3012 Bern, Switzerland; joachim.mueller@unibe.ch (J.M.); anitha.vigneswaran@students.unibe.ch (A.V.); kai.haenggeli@unibe.ch (K.P.A.H.); yosra.amdouni@unibe.ch (Y.A.); 3 Department of Chemistry, Life Sciences and Environmental Sustainability, University of Parma, Parco Area delle Scienze, 11/a, 43124 Parma, Italy; mirco.scaccaglia@unipr.it (M.S.); giorgio.pelosi@unipr.it (G.P.); franco.bisceglie@unipr.it (F.B.); 4 Proteomics and Mass Spectrometry Core Facility, Department for BioMedical Research (DBMR), University of Bern, Länggass-Strasse 122, 3012 Bern, Switzerland; manfred.heller@dbmr.unibe.ch (M.H.); anne-christine.uldry@dbmr.unibe.ch (A.-C.U.); pmscf@dbmr.unibe.ch (S.B.-L.)

**Keywords:** *Toxoplasma gondii*, thiosemicarbazone, drug screening, ribosomal proteins, affinity chromatography, proteomics, drug tolerance

## Abstract

Thiosemicarbazones and their metal complexes have been studied for their biological activities against bacteria, cancer cells and protozoa. Short-term in vitro treatment with one gold (III) complex (C3) and its salicyl-thiosemicarbazone ligand (C4) selectively inhibited proliferation of *T. gondii*. Transmission Electron Microscopy (TEM) detected transient structural alterations in the parasitophorous vacuole membrane and the tachyzoite cytoplasm, but the mitochondrial membrane potential appeared unaffected by these compounds. Proteins potentially interacting with C3 and C4 were identified using differential affinity chromatography coupled with mass spectrometry (DAC-MS). Moreover, long-term in vitro treatment was performed to investigate parasitostatic or parasiticidal activity of the compounds. DAC-MS identified 50 ribosomal proteins binding both compounds, and continuous drug treatments for up to 6 days caused the loss of efficacy. Parasite tolerance to both compounds was, however, rapidly lost in their absence and regained shortly after re-exposure. Proteome analyses of six *T. gondii* ME49 clones adapted to C3 and C4 compared to the non-adapted wildtype revealed overexpression of ribosomal proteins, of two transmembrane proteins involved in exocytosis and of an alpha/beta hydrolase fold domain-containing protein. Results suggest that C3 and C4 may interfere with protein biosynthesis and that adaptation may be associated with the upregulated expression of tachyzoite transmembrane proteins and transporters, suggesting that the in vitro drug tolerance in *T. gondii* might be due to reversible, non-drug specific stress-responses mediated by phenotypic plasticity.

## 1. Introduction

*Toxoplasma gondii* (*T. gondii*) is a globally distributed, apicomplexan parasite and is responsible for one of the most important parasitic zoonoses worldwide [1,2,3]. In healthy individuals, infection with *T. gondii* is often asymptomatic or mild but can have severe consequences in pregnant women and immunocompromised individuals. Following primary infection during pregnancy, *T. gondii* may be transmitted to the developing fetus, resulting in congenital toxoplasmosis. Spontaneous abortion may occur, or the neonate may present with bilateral chorioretinitis, intracranial calcifications, hydrocephalus or microcephaly. Clinical manifestations, including ocular disease, may appear months or years following congenital infection [4]. In immunocompromised individuals with chronic infection (for example AIDS/HIV+ patients or individuals undergoing treatment with immunosuppressive drugs), *T. gondii* tissue cysts can reactivate, and parasites can be released and resume proliferation [3]. This can lead to severe toxoplasmosis with a potentially fatal outcome [5]. The most frequently used drugs for the treatment of toxoplasmosis are a combination of sulfadiazine and pyrimethamine [6,7], which inhibit enzymes involved in folate biosynthesis. Other treatment options include clindamycin or clarithromycin combined with pyrimethamine, or quinolones such as atovaquone. However, these drugs are not effective in all cases, can be associated with marked side effects and are not able to eliminate the tissue cysts that are responsible for chronic infection [8,9]. Thiosemicarbazones (TSCs) are organosulfur compounds which have been shown to exhibit antimicrobial and anticancer activity [10,11,12,13]. Two TSC iron chelators of the di-2-pyridylketone thiosemicarbazone (DpT) class, Dp44mT and DpC, were highly effective against cancer cells in vitro and in vivo, and were thought to accumulate in the lysosomes, leading to the generation of reactive oxygen species (ROS) and induction of apoptosis [14]. Another TSC cancer drug, triapine (3-aminopyridine-2-carboxaldehyde- thiosemicarbazone), was reported to cause cell death by inducing mitochondrial alterations that enhance ROS production [15]. Since 2005, over fifty TSC derivatives have been evaluated for activity against *T. gondii* in vitro, and several compounds exhibited a promising potential against the proliferative tachyzoite stage [16,17,18,19,20,21,22]. A study evaluating 4-arylthiosemicarbazides suggested that the TSC scaffold acted on *T. gondii* tyrosine metabolism through inhibition of the tyrosine hydroxylase, leading to inhibition of proliferation in vitro [17]. Whether parasites could adapt or develop tolerance to these compounds, however, has not yet been investigated. Gold-based complexes have gained particular attention for the treatment of *T. gondii* infection. A classic example is auranofin, which is FDA-approved for the treatment of rheumatoid arthritis [23,24] and was repurposed as an antibacterial compound [24] and also shown to be highly efficacious against *T. gondii*, both in vitro and in an experimental model of acute toxoplasmosis [25]. Gold (III) complexes bearing pyridine, porphyrin, phosphines and TSC ligands were synthesized [26,27] and have been suggested to target cysteine or seleno-cysteine containing enzymes such as thioredoxin reductase, phosphatases and cathepsin, all of which are present in *T. gondii* [28,29]. Also, depending on their ligand, some gold (III) complexes were shown to interact with genomic DNA [30].

**Figure 1:**
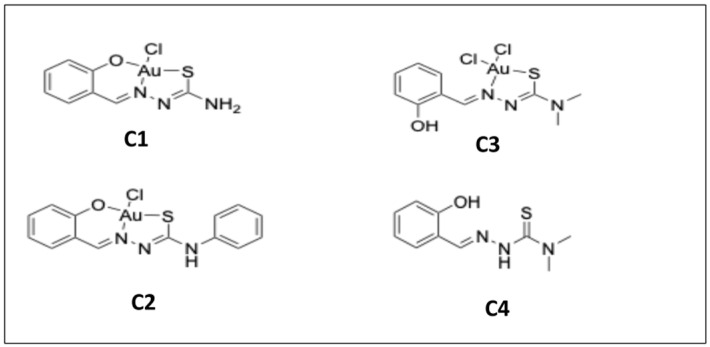
Chemical structures of three gold (III) complexes and their thiosemicarbazone ligand. **C1**: salicylaldehyde thiosemicarbazone gold (III) chloride, **C2**: salicylaldehyde 4,4-Dimethyl-3-thiosemicarbazide gold (III) dichloride, **C3**: salicylaldehyde 4-phenylthiosemicarbazide gold (III) chloride, and **C4**: salicylaldehyde 4,4-dimethyl-3-thiosemicarbazide.

In the present study, three gold (III) complexes with salicyl-TSC ligands (Figure 1) were assessed for activity against *T. gondii* and human foreskin fibroblast (HFF) host cells. One gold complex (named C3) and its corresponding salicyl-TSC ligand (named C4) exhibited promising inhibition constants and were further studied. Interestingly, *T. gondii* tachyzoites rapidly adapted to these compounds within a few days of drug treatment. Thus, the inhibition by C3 and C4, as well as the adaptation of *T. gondii* to these drugs, was of a transient nature. To investigate this phenomenon, differential affinity chromatography coupled to mass spectrometry and proteomics (DAC-MS-proteomics) was applied to *Toxoplasma* tachyzoite protein(s) binding to C4 and C3, and differential protein expression profiles of six clones of *T. gondii* tachyzoites adapted to C3 and C4 were analyzed in comparison to non-adapted *T. gondii* tachyzoites. This is one of the rare studies combining target deconvolution and investigation of laboratory generated adapted strains of the same genetic background.

## 2. Results

### 2.1. In Vitro Activities of Three Gold Complexes and Their Salicyl-TSC Ligand (C4) against T. gondii-tachyzoites

Of the four compounds shown in Figure 1, C1 and C2 were toxic for HFF and thus also prevented the replication of *T. gondii*, and therefore no selective toxicity could be measured (Supplementary Figure S1). On the other hand, the gold (III) complex C3 and its salicyl-TSC ligand C4 exhibited low *T. gondii* β-galactosidase (β-Gal) half maximal effective concentration (EC_50_) values ranging between 20 and 30 nM, whether the compounds were added concomitantly or 3 h after host cell invasion (see Table 1).

*T. gondii* β-Gal is a transgenic type I strain with RH background. EC_50_ values were also determined for type II *T. gondii* ME49 tachyzoites by quantitative real-time PCR, and results obtained with both compounds were comparable to results obtained with *T. gondii*-β-Gal (Table 1). Alamar blue assay showed no impairment of viability of non-infected HFF monolayers at concentrations up to 25 μM (Table 1).

### 2.2. C3 and C4 Induce Only Minor and Transient Ultrastructural Alterations in and Do Not Affect the Mitochondrial Membrane Potential T. gondii ME49 Tachyzoites

**Figure 2:**
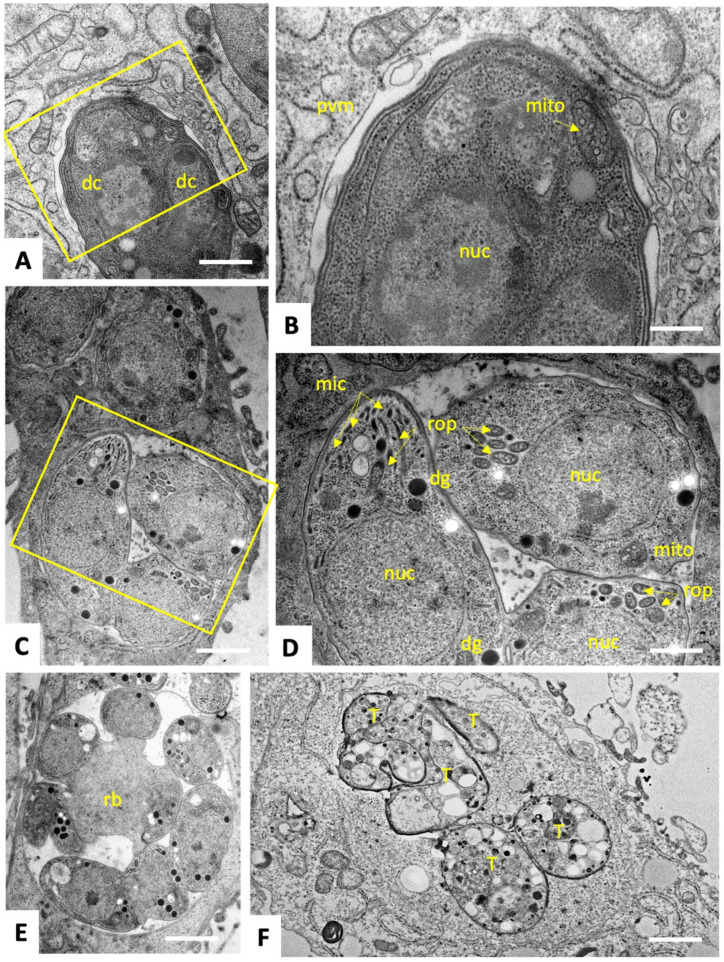
TEM of non-treated *T. gondii* ME49 tachyzoites (**A**–**E**) and tachyzoites treated with the positive control drug DB745. (**A**) A tachyzoite undergoing endodyogeny with the two emerging daughter cells (dc) at 12 h post-infection, situated within a parasitophorous vacuole. The yellow boxed area in (**A**) is shown at higher magnification in (**B**). The vacuole is surrounded by the parasitophorous vacuole membrane (pvm). (**C**) A vacuole containing numerous tachyzoites at 24 h p.i., the yellow boxed area is shown at higher magnification in (**D**), revealing rhoptries. (rop), dense granule (dg), micronemes (mic), the mitochondrion, and the nucleus (nuc), all of them indicated with yellow arrows. (**E**) Proliferating tachyzoites still attached to a residual body (rb) within a parasitophorous vacuole at 48 h of culture. (**F**) tachyzoites treated with DB745 during 48 h, showing tachyzoites (yellow T in the figure) with severe structural alterations. Bars in (**A**) = 1 µm; (**B**) = 0.3 µm; (**C**) = 1.2 µm; (**D**) = 0.6 µm; (**E**) = 1.5 µm; (**F**) = 1.2 µm.

**Figure 3:**
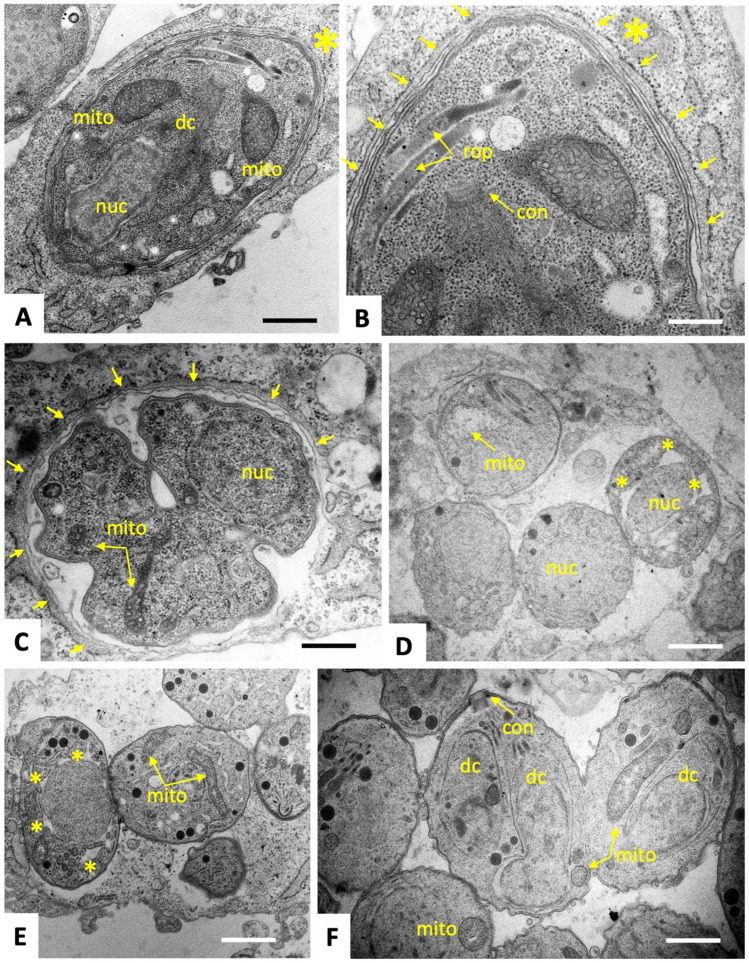
TEM of *T. gondii* ME49 tachyzoites grown in HFF and treated with 0.5 µM C3 during 6–12 h (**A**–**C**), 24 h (**D**,**E**) and 48 h (**F**). A tachyzoite undergoing endodyogeny with an emerging daughter cell (dc) after 6 h of treatment is depicted in (**A**) and the respective apical part marked with an asterisk (*), including the conoid of the daughter cell (con) shown at higher magnification in (**B**). A large portion of the parasitophorous vacuole is surrounded by a multi-layered membrane (marked with small yellow arrows). Similar findings were obtained after 12 h (**C**). At 24 h, the multilayered membrane surrounding the parasitophorous vacuole was not visible anymore, but many parasites exhibited cytoplasmic alterations, leaving an empty space between the nuclear periphery and the cytoplasm, marked by asterisks (*) as shown in (**D**,**E**), and the matrix of the mitochondrion (mito) was partially dissolved. At 48 h, most tachyzoites had lost these ultrastructural alterations and had a normal appearance (**F**). Bars in (**A**) = 0.8 µm; (**B**) = 0.5 µm; (**C**) = 0.8 µm; (**D**) = 1 µm; (**E**) = 1 µm; (**F**) = 0.8 µm.

**Figure 4:**
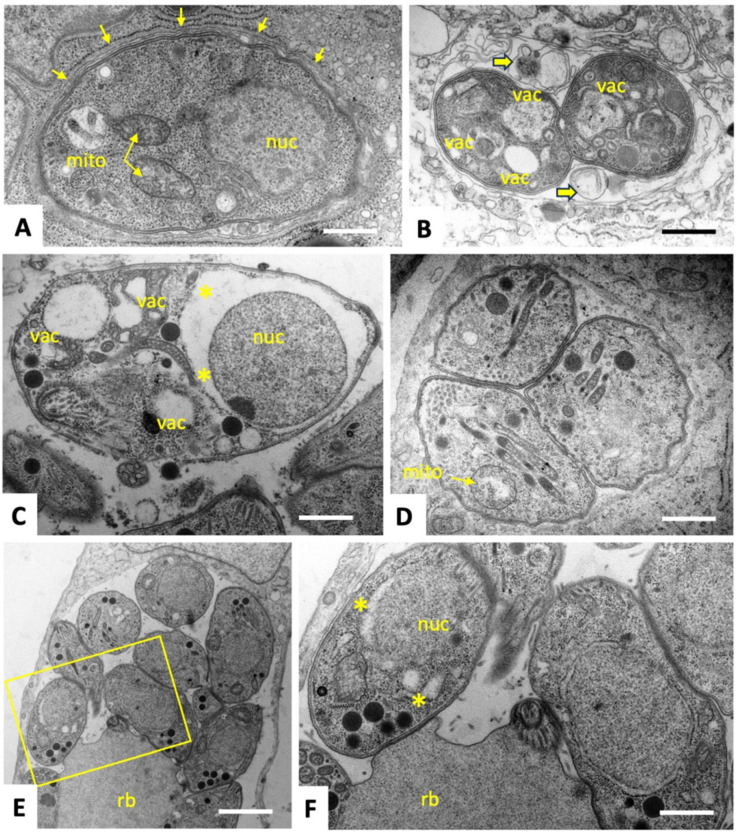
TEM of *T. gondii* ME49 tachyzoites grown in HFF and treated with 0.5 µM of C4 during 6 h (**A**), 12 h (**B**,**C**), 24 h (**D**), and 48 h (**E**,**F**). The yellow boxed area in (**E**) is depicted at higher magnification in (**F**). A multi-layered membrane, indicated by small arrows surrounding the parasitophorous vacuole is evident after 6 h of treatment (**A**). After 12 h of treatment (**B**,**C**), ultrastructural alterations became visible in a large number of parasites as evidenced by the accumulation of membranous components within the vacuolar space (showed with thick yellow arrows) and increased numbers of cytoplasmic vacuoles (vac), and in some parasites, a separation of the nucleus from the surrounding cytoplasm was visible (asterisks *, see (**C**)). In addition, pronounced alterations of the mitochondrial matrix (mito) were noted after 24 h (**D**). At 48 h after initiation of drug treatment (**E**,**F**), parasites exhibited a largely normal structural appearance, with the exception of some parasites still maintaining free spaces between nuclear membrane and cytoplasm (asterisks *). Bars in (**A**) = 0.6 µm; (**B**) = 1 µm; (**C**) = 0.5 µm; (**D**) = 0.9 µm; (**E**) = 2.2 µm; (**F**) = 0.9 µm.

To characterize ultrastructural changes in tachyzoites exposed to C3 and C4, treated and untreated parasites were comparatively analyzed by transmission electron microscopy (TEM). *T. gondii* ME49 tachyzoites cultured in the absence of compounds (Figure 2) underwent intracellular proliferation by endodyogeny within a parasitophorous vacuole, separated from the cytoplasm by a parasitophorous vacuole membrane (PVM). Tachyzoites exhibited the typical hallmarks of apicomplexans including secretory organelles such as rhoptries, micronemes and dense granules, and a conoid situated at the apical pole. Tachyzoites harbor a single mitochondrion composed of branched tubules, of which only parts were visible in any given section plane, exhibiting densely packed cristae within an electron dense matrix. Normally, parasites proliferate by endodyogeny, and in rapidly dividing cultures multiple newly formed tachyzoites were often seen emerging from a residual body. At 6–12 h after initiation of drug treatments with C3 (Figure 3) and C4 (Figure 4), few but distinct changes were detectable. For instance, a large portion of parasitophorous vacuoles (>50%) were surrounded by a multi-layered parasitophorous vacuole membrane (Figure 3A–C and Figure 4A), as opposed to the typical single membrane surrounding non-treated parasites. At these early time points, tachyzoites in C3-treated cultures, however, did not appear to be structurally altered and underwent cell division, while approximately 30% of parasites in C4-treated cultures exhibited an aberrant cytoplasmic organization, most notably with increased vacuolization and “empty spaces” forming between the nuclear membrane and the cytoplasm of tachyzoites (Figure 4C,D). At 24 h of C3 treatment, such cytoplasmic alterations were also visible occasionally in 10–20% of tachyzoites, (Figure 3D,E), and slight structural disturbances such as a partially dissolved matrix could be detected in the mitochondrion in C3-treated (Figure 3D) and C4-treated (Figure 4D) tachyzoites. However, no effect on the mitochondrial membrane potential could be seen when assessed by tetramethylrhodamine ethyl ester uptake assay (TMRE) (Supplementary Figure S2). At 48 h, C3-treated parasites had largely recovered, and only slight alterations could be seen in some (<5%) of the tachyzoites in C4-treated cultures. In conclusion, the changes induced by treatments with C3 and C4 were rather mild and apparently only of a transient nature.

### 2.3. Identification of C3- and C4-Binding Proteins in Extracts of T. gondii ME49 tachyzoites

**Figure 5:**
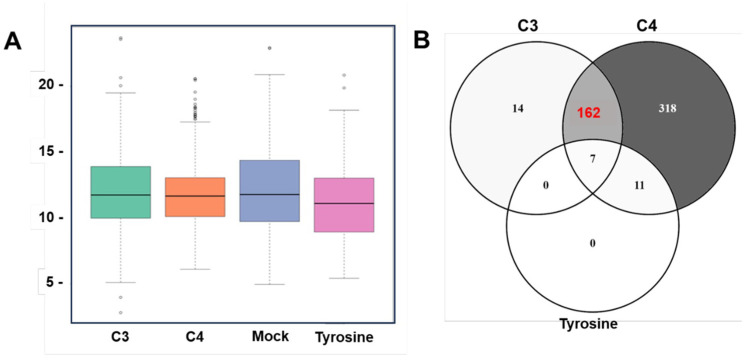
Identification of *Toxoplasma* proteins that bind to C3 and/or C4 by affinity chromatography coupled mass spectrometry. (**A**): box and whisker plot describing protein intensity distributions as calculated by the iBAQ (intensity Based Absolute Quantification) algorithm across eluates from C3 (gold (III) complex), C4 (salicyl-TSC ligand), mock and tyrosine columns. (**B**): Venn diagram detailing the distribution of identified proteins (512) which were not binding to the mock column.

To characterize proteins that bind specifically to C3 and C4, and to both compounds, differential affinity chromatography (DAC) was combined with mass spectrometry (MS)-based quantitative proteomics of respective eluates. Besides C3 and C4, we included tyrosine as a control compound having a closely related chemical structure, but with no effects on *T. gondii* proliferation. Overall, the analysis of the DAC eluates yielded 5137 unique peptides that were mapped to 630 *T. gondii* proteins (see Supplementary Table S1 for the full dataset) with equal intensity distribution between samples as displayed in Figure 5A.

A total of 118 proteins were exclusively identified in the mock-column eluate, whereas 512 proteins were found in proteomes obtained from eluates of the remaining three columns: C3, C4 and tyrosine. The largest proportion of the identified proteins (318/630) were exclusively C4-binding proteins, whereas only 14 proteins were exclusively C3-binding proteins (Figure 5B). One hundred sixty-two proteins were commonly identified in proteomes obtained from C3 and C4 columns, while only seven proteins were commonly found in the eluates of tyrosine, C3 and C4 columns (Figure 5B). The fourteen proteins exclusively binding to the C3 column are shown in Table 2 as ranked by relative abundance. The ribosomal protein RPL27 (TGME49_262690) was the most abundant and constituted 42% of total C3-specific binding proteome (Table 2). Among the 318 proteins specifically binding to C4, the SAG-related sequence (SRS) protein SRS25 encoded by ORF TGME49_213280 was the most abundant (Table 3). A total of 11 SRS proteins were identified as specifically binding to C4, five of which were among the 20 most abundant proteins (Table 3). The hypothetical protein encoded by ORF TGME49_253690 was the second most abundant protein binding to C4 (Table 3). The ribosomal protein RPSA (ToxoDB ORF: TGME49_266060) was among the five most abundant proteins and represented 3% of the C4-specific binding proteome. A large portion (50/162; 31%) of the 162 proteins that did bind to both C3 and C4 but were not found in mock or tyrosine eluates were ribosomal proteins. In Table 4, the twenty most abundant proteins which are shared between C3 and C4 columns are ranked according to their relative abundance. Most (18 out of 20) proteins commonly interacting with C3 and C4 were ribosomal proteins (Table 4).

### 2.4. Long-Term Treatments and Generation of Clones of Drug-Adapted T. gondii Tachyzoites

Further studies were carried out using *T. gondii* ME49 tachyzoites. While C3 and C4 treatments were highly effective in short-term three-day-growth assays, treatments with both compounds at 0.5 µM for an additional four to eight days revealed that parasites quickly resumed proliferation despite continuous drug pressure. To investigate the actual drug susceptibility of such drug-adapted strains, C3- and C4-adapted tachyzoites were cloned, and three clones/drugs were frozen as stabilates and stored at −150 °C. Subsequently, the stabilates were thawed and subjected to EC_50_ and minimum inhibitory concentration (MIC) determinations and results are summarized in Table 5 and Supplementary Figure S3. For both drugs, the EC_50_ values for the *T. gondii* ME49 tachyzoites representing the wildtype (WT) strain and the C3- and C4-adapted strains (three clones each) were in the similar range, suggesting that the effect was lost after freeze/thawing and that parasites had only transiently adapted to increased drug concentrations (Table 5). MIC values were in the same range (25 µM) for all strains tested. Taken together, these findings indicate that *T. gondii* did not develop in vitro resistance against C3 or C4, but only transiently adapted to the environmental stress induced by C3 and C4, through a mechanism that was reversible.

### 2.5. Proteomic Analysis of T. gondii ME49 Clones Recovered after In Vitro Adaptation to C3 and C4

The *T. gondii* ME49 WT strain and the six clones derived from C3- and C4-treated cultures (three clones per drug) were analyzed by whole cell shotgun proteomics to identify differentially expressed (DE) proteins. As shown in Supplementary Table S2, a total of 3860 proteins were identified. To reliably identify differentially expressed proteins in C3- and/or C4-treated tachyzoites versus non-treated WT, we considered as significantly up- and down-regulated proteins in treated parasites compared to WT only those with fold change FC ≥ 1.5 (Log2FC ≥ 0.58) and those with FC ≤ 0.66 (Log2FC ≤ −0.58), respectively. Thus, a total of 131 and 72 proteins were found to be differentially expressed in C3- and C4-treated cultures, respectively, compared to WT parasites. C3-clones showed upregulation of the expression of 74 proteins and downregulation of 57 proteins compared to WT tachyzoites (Supplementary Table S2). Most of the dysregulated proteins (125/131) upon treatment with C3 were found exclusively in clone P1E5. Two proteins (TGME49_251180 and TGME49_229140) were commonly dysregulated in the three C3-clones P1F3, P1E5 and P2F5, whereas the ORF TGME49_238200 encoding for alpha/beta hydrolase fold domain-containing protein was shared between P2F5/P1E5 (Supplementary Table S2). Table 6 presents the top 20 up- and downregulated proteins in C3-clones, ranked according to their FC. Interestingly, the upregulated ribosomal protein RPL44 encoded by ORF TGME49_203630 was also identified as a C3-binding protein (Supplementary Table S1). Another upregulated protein (FC = 1.7 in P1E5/WT, see Supplementary Table S2) annotated as cyst wall protein CST7 (TGME49_258870) shown in Supplementary Table S2, was also identified as a C3-binding protein (Supplementary Table S1). The most downregulated proteins in C3-treated clones were the putative ribosomal RNA (adenine(1779)-N(6)/adenine(1780)-N(6))dimethyltransferase encoded by the ORF TGME49_248200, and the cyst matrix protein MAG2 (TGME49_209755) (Table 6). Proteomic analyses of clones recovered from C4 treatment compared to *T. gondii* ME49 WT parasites showed that 66 proteins were upregulated (FC ≥ 1.5 (Log2FC ≥ 0.58); *p* ≤ 0.05) and six proteins were downregulated (FC ≤ 0.66; *p* ≤ 0.05). As shown in Table 7, the expression of hypothetical protein encoded by ORF TGME49_306300 was more than seven-fold higher in clone P1G10 compared to *T. gondii* ME49 WT parasites. TGME49_306300 contains a conserved domain (TIGR00927) described as a K+-dependent Na+/Ca+ exchanger, which is reminiscent of transport and binding proteins for cations and iron carrying compounds. Three other upregulated proteins, namely the hypothetical protein (TGME49_277920), the ribosomal protein RPS27 (TGME49_217570) and the ribosomal-ubiquitin protein RPS27A (TGME49_245620) (Supplementary Table S2) were also identified as C4-binding proteins by C4-affinity chromatography (Supplementary Table S1), and among those the ribosomal protein RPS27A was also identified in the C3-binding fraction by affinity chromatography (Supplementary Table S1). A further 11 upregulated proteins and one downregulated protein were identified in both C3- and C4-recovered parasites, in comparison to WT tachyzoites. As shown in Table 8, those proteins exhibiting the highest upregulated expression are transmembrane proteins and membrane transporters.

## 3. Discussion

In the present study, three gold (III)–salicyl-TSC complexes (C1-3) and one salicyl-TSC compound (C4) were assessed for in vitro activity against *T. gondii* tachyzoites. C4 is the respective ligand of C3. C3 and C4 exhibited specific toxicity against *T. gondii* tachyzoites with EC_50_ values below 50 nM and no impairment of HFF viability at a > 500 times higher concentration. The fact that the salicyl-TSC ligand (C4) by itself was as active as the respective C3 gold (III) complex indicates that the presence of gold was not necessary to exert the anti-parasitic effect, and this is in agreement with previous studies demonstrating anti-protozoal activity for thiosemicarbazones, including *T. gondii* and *T. cruzi* [17,31]. Similar efficacies of the gold (III) complex C3 and its ligand C4 were noted upon the addition of the drugs concomitantly to infection or after parasites were already intracellular, which suggests that these drugs affect intracellular parasites rather than inhibiting *T. gondii* tachyzoite entry into host cells. TEM of treated versus non-treated *T. gondii* ME49 tachyzoites was performed to investigate how intracellular tachyzoites were affected by C3 and C4 exposure. Surprisingly, some effects were observed only at the early stages of treatment (6–24 h) and were slightly more pronounced upon C4 treatment. Common features seen for both C3 and C4 treatments were the build-up of several layers of membranes surrounding the parasitophorous vacuole and moderate cytoplasmic vacuolization, as well as distinct alterations in the matrix of the parasite mitochondrion. It is not clear whether the additional parasitophorous vacuole membrane material is generated by the parasite, or whether it is derived from the host. However, as there is currently no mechanism known by which *T. gondii* could form additional lipid bilayer membranes, it is conceivable that these membranes are host cell derived. This would imply that they lack *T. gondii* transporter proteins to acquire nutrients from the host cytoplasm, which in turn could be partially responsible for the observed cytoplasmic vacuolization as well as mitochondrial alterations. Thus, the effects of the drugs could be not directly on the tachyzoites but could be mediated, at least partially, by the host cell, potentially as attempted autophagy. This would also explain why the effect of the drug is restricted to intracellular tachyzoites. It is important to note that (i) these aberrant ultrastructural alterations were not found in all but in a majority of tachyzoites, (ii) they were not evident in the controls; the alterations occurred only transiently, with most parasites returning to their original ultrastructural characteristics after 48 h. Thus, we cannot exclude the possibility that the tachyzoites showing these ultrastructural alterations die and disappear (possibly by authophagy by the host cells) and that the non-affected tachyzoites are only responsible for the observed growth during longer-term treatments. In order to identify potential interaction partners of C3 and C4 in *T. gondii*, differential affinity chromatography coupled with mass spectrometry was performed. Since ribosomal proteins, with 31% relative abundance, represent by far the major proportion of C3- and C4-binding proteins, these compounds may interfere with protein biosynthesis. In cancer cells, thiosemicarbazones, particularly those presenting a thio carbonyl (C=S) bond, have been shown to interfere specifically with the function of RLP44 during the elongation step of translation by inhibiting the crosslinking reaction between RPL44 and the periodate-oxidized tRNA [32]. Translation is one of the best investigated targets of antimicrobials. The first drugs against toxoplasmosis included antibiotics that inhibit translation, including clindamycin [33] and spiramycin [34]. In addition, affinity chromatography identified several quinoline-binding proteins in the closely related *Neospora caninum* that are involved in translation [35]. If inhibition of translation is the real mode of action, the effect appears to be reversible, since prolonged treatment resulted in adaptation of *T. gondii* tachyzoites to drug exposure and parasites subsequently resumed proliferation. Results from comparative examination of proteomic data from *T. gondii* ME49 WT and the six clones recovered from C3 and C4 treatments suggest that there are three main strategies of molecular mechanisms by which tachyzoites could reverse the effect of C3- and C4-salicyl-TSC based drugs. A first strategy could be the overexpression of C3 and C4 drug targets to restore their normal levels. This is largely supported by the STRING protein–protein interaction network analysis of upregulated proteins (see Supplementary Figure S4) which reveals a cluster of ribosomal proteins. In contrast, the STRING analysis of downregulated proteins identified a network consisting of proteins involved in DNA replication and repair (see Supplementary Figure S5), which then may explain the initial inhibition of proliferation seen in the presence of the compounds. A significant example of the target overexpression strategy is given by the ribosomal protein RPL44, whose expression level increased in all six clones compared to WT tachyzoites. In addition, the expression of RPS27, which binds to both C3 and C4 by affinity chromatography, was increased in C4-adapted clones. Binding of this and various other ribosomal proteins to the compounds suggests that they target the ribosomes and possibly interfere with protein biosynthesis. The standard procedure to confirm drug targets consists in generating either knockouts or overexpressing strains and testing their EC_50_ values in comparison to the parental cell lines. Since protein biosynthesis is essential, it is obvious that this approach cannot be performed in this special case. Moreover, the selection procedure to obtain genetically modified strains may interfere by itself with gene expression [36]. It is also important to keep in mind that just because a compound can bind to a solubilized ribosomal protein in isolation, it may not have access to that same binding motif when the protein is part of the assembled ribosome, or that it would inhibit ribosomal functions. However, for spectinomycin, which is a ribosome-targeting antibiotic, it has also been shown that bacteria enhance the expression of the 16S rRNA helix 34, which is the main target. The 16S rRNA helix 34 is, on one hand, needed for translation, but it will also sequester the drug when overexpressed [37]. A second possible strategy identified in the present study for developing tolerance to C3 and C4 is the upregulation of cellular efflux transporter proteins, which could lead to a reduction of intracellular C3 and C4 concentrations. The three proteins with the highest level of upregulation in adapted strains as compared to their WT, namely the hypothetical protein encoded by TGME49_306300, a putative transporter encoded by TGME49_258700 and another hypothetical protein encoded by TGME49_248140 containing two short transmembrane domains may be involved in this strategy. However, these proteins have not been functionally characterized with respect to transport of xenobiotics so far, and exact localization studies should be carried out to verify the localization of these proteins at the plasma membrane. In addition, another protein, the soluble N-ethylmaleimide-sensitive-factor attachment protein receptor (SNARE) protein STX20 (TGME49_253360), was upregulated in five out of the six analyzed clones. In *T. gondii*, SNAREs are involved in exocytosis at the plasma membrane [38], and it has recently been shown that *T. gondii* STX20 associates with STX1 and STX21 to form an unconventional SNARE complex, which mediates exocytosis at the plasma membrane and vesicular fusion at the apical annuli [39]. Furthermore, it is well known that bacteria upregulate the expression of porins and efflux pumps in response to most ribosome-targeting antibiotics [40,41,42]. Thirdly, our proteomics analyses indicate that *T. gondii* responded to C3 and C4 treatments by metabolization, degradation or modification of C3 and C4, e.g., via upregulated oxidases, dehydrogenases or hydroxylases, similar to that previously reported for tetracycline [43]. Four of the six clones adapted to either C3 or C4 exhibited an upregulated expression of the alpha/beta hydrolase fold domain-containing protein (TGME49_238200). The functions of these enzymes in *T. gondii* are, however, unknown. Rapid adaption of *T. gondii* to elevated drug concentrations in vitro has also been described for other compounds, including decoquinate- and artemisinin-derivatives [44,45], bumped kinase inhibitors [46], ruthenium-based compounds [47] and pentamidine derivatives [48], and has also been reported in other apicomplexans including *N. caninum* tachyzoites treated with endochin-like quinolones [49] and *Besnoitia besnoiti* tachyzoites treated with buparvaquone. Overall, this suggests that the emergence of adapted/resistant parasites following long-term drug treatment in vitro is not uncommon, as has also previously occurred with sulfonamides [50,51], although the mechanism of decreased drug susceptibility in the latter case was based on mutations in the gene sequences that encode the drug target, specifically enzymes involved in the folate pathway. Similar EC_50_ and MIC values were determined for both compounds using clones recovered after C3 and C4 adaptation and regrowth after freezing, as well as for WT *T. gondii*. Results showed that adaptation to these drugs was only a transient feature and was lost in absence of drugs after one cycle of cryopreservation. Consequently, the six clones regained similar drug susceptibilities as the WT parasites but could quickly readjust once drugs were added back to the culture medium. These results highlight the considerable phenotypic plasticity in *T. gondii*, which is often mediated by epigenetic changes, allowing the parasite to respond, in time, to environmental stress, including drugs [52]. Moreover, differentially expressed proteins of clone P1E5 belonging to the epigenetic machinery [53] such as the SWI2/SNF2-containing protein (TGME49_273780) and the lysine-methyltransferase encoded by TGME49_281900 suggest the involvement of epigenetic mechanisms in the adaptation of this clone. The fact that the repressor of bradyzoite-specific genes ApiAP2IV-4 (TGME49_318470) [54] is one of the upregulated proteins of the same clone indicates a link between developmental regulation and drug adaptation. Furthermore, the fact that varying differentially expressed protein patterns are found in different clones suggests alternative modes of adaptation which are equally effective, similar to what has been observed in different strains of nitro drug-resistant *Giardia lamblia* strains [55]. Interestingly, a higher number of differentially expressed proteins were identified in clones resistant to C3 than in clones resistant to C4. This may be due to the presence of a gold ion-causing oxidative stress and thereby stimulating a variety of responses such as transport or metabolization [56,57]. Moreover, the degree of dysregulation of SAG1-related sequence (SRS) proteins varied among C3 and C4 clones, and the list of SRSs binding to C3 and/or C4 in affinity chromatography did not correlate with the list of differentially expressed SRSs. In fact, eleven SRS proteins were identified in affinity chromatography eluates from C4 columns, with SRS25 being the most abundant protein binding to C4. However, none of these SRSs showed a changed expression in C4-derived clones. In contrast, none of the SRSs was identified among proteins binding to C3, while a cluster of six SRSs was differentially expressed in C3-adapted clones with 5/6 being downregulated. Taken together, these results suggest that SRS25 binds to C4 but is not acting as a target, and that the differential expression of the six SRSs in clones recovered from C3 treatment is likely an arbitrary event. This hypothesis is largely supported by the fact that SRS expression patterns in in vitro maintained tachyzoites is rather random [58]. Consistent with this, we also found a significant difference in SRS expression patterns between individual SRS29B knock-out clones of *T. gondii* RH [36]. In conclusion, our results strongly suggest that the gold (III) complex (C3) and its salicyl-TSC ligand (C4) interfere with translation in *T. gondii* by targeting ribosomal proteins. The rapid adaptation to salicyl-TSC based drugs is likely mediated by different mechanisms such as upregulation of some of the target proteins, enhanced cellular efflux function and possible drug inactivation/metabolism rather than by downregulation of targets essential for cellular maintenance. Adaptation, however, was reversible, and tolerance to these drugs was rapidly lost in their absence. The phenomenon described herein is distinct from drug resistance, which implies either introducing mutations in a drug target or stable epigenetic changes altering the expression. Nevertheless, this transient adaptation illustrates how these intracellular parasites can rapidly deal with environmental changes and displays the outstanding plasticity of the gene expression pattern of *T. gondii.* Future studies should determine whether related mechanisms could apply to other TSCs and other drug classes that have shown inhibitory effects on parasite survival. DAC and proteomic analysis of drug-treated parasites undertaken here represent an interesting starting point, and further validation of the proposed mechanisms is needed in the future to dissect this transient adaptive feature displayed by *T. gondii* tachyzoites. We conclude that *T. gondii* is an excellent model system to perform target deconvolution studies and analyses of adapted or resistant strains in parallel and with the same genetic background. Such studies are scarce and merit greater attention.

## 4. Materials and Methods

### 4.1. Equipment and Reagents Used in Cell Culture

Unless otherwise specified, all cell culture devices were purchased from Sarstedt (Sevelen, Switzerland), media from Gibco-BRL (Zürich, Switzerland) and biochemicals from Sigma (St. Louis, MO, USA).

### 4.2. Compounds

Three gold (III) complexes and their thiosemicarbazone ligand (Figure 1) were synthesized according to Scaccaglia et al. [57]. Briefly, the ligands were synthesized by reacting the corresponding aldehyde with thiosemicarbazides in an alcohol solution. The resulting precipitate was filtered and employed in the synthesis of gold (III) compounds through a reaction with HAuCl4.

The stability of the gold (III) complexes over 24 h at 37 °C was checked by UV-Vis spectroscopy using the RPMI cell culture medium to mimic physiological conditions over time. No significant variation in the absorption profile was detected, indicating that the ligands are suitable to stabilize gold (III) under these conditions [57].

For in vitro testing, drug stock solutions were prepared at 20 mM concentration in dimethyl sulfoxide (DMSO) and stored at −20 °C.

### 4.3. Host Cells and Parasites

Human foreskin fibroblasts (HFF; _PCS-201-010TM, American Type Culture Collection, Manassas, VA 20110-2209, USA) were cultured as previously described [59]. For in vitro assessment of the synthetized compounds, we used two *Toxoplasma gondii* strains: (i) *T. gondii* β-gal (type I), a genetically modified strain constitutively expressing β-galactosidase kindly provided by Prof. David Sibley (Washington University, St. Louis, MO, USA), and (ii) *T. gondii* ME49 (type II), a strain which was provided by Dr. Furio Spano (Istituto Superiore di Sanità, Roma, Italy). Tachyzoites of both strains were maintained in vitro as described earlier [59].

### 4.4. In Vitro Assessment of Toxicity and Efficacy of Compounds in HFF and T. gondii tachyzoites

Cytotoxicity was assessed using uninfected HFFs and determining the EC_50_ as described earlier [60]. Briefly, HFFs were seeded in 96-well plates at a density of 5 × 10^3^ cells/well until the cells reach 80% confluency. Serial dilutions (ratio 1:2) of the compounds were prepared (50 µM–0.8 µM) and added to HFF cultures. After culture for 72 h at 37 °C/5% CO_2_, the medium was discarded, and viability of HFF was determined by Alamar blue assay as previously described [60]. Drug concentrations that led to 50% reduction in cell viability as compared to fully viable controls (set to 100%) were calculated using the logit-log algorithm.

Susceptibility of *T. gondii* β-gal to the tested compounds was assessed by determining EC_50_ values based on a β- galactosidase assay as described earlier [60]. Serial dilutions (ratio 1:2) of compounds (10 μM to 1 × 10^−5^ µM) were prepared and added either 5 min prior to infection of host cells (EC_50_ in pre-infection in vitro model) or 3 h after infection (EC_50_ in post-infection in vitro model, for C3 and C4). For determining EC_50_ values of C3- and C4-adapted and WT-*T. gondii* ME49 tachyzoites, confluent HFF monolayers grown in 24-well plates received drugs diluted in Dulbecco’s Modified Eagle Medium (DMEM) complemented with Fetal Bovine Serum (FBS) containing drug concentrations ranging from 4 × 10^−3^ to 2 µM, and were infected with 2 × 10^3^ freshly purified tachyzoites in a total volume of one ml. After five days of culture at 37 °C/5%CO_2_, the culture medium of each well was collected and centrifuged at 2000× *g* for 10 min, supernatants were discarded and pellets collected. In addition, all cellular material in each well was scraped and lysed in 250 µL of RNA Lysis Buffer (RLY) containing proteinase K (Nucleospin Rapid lyse kit, Macherey-Nagel, Düren, Germany). The pellets from the supernatants and the corresponding lysates from the wells were combined in Eppendorf tubes and DNA was extracted using the Nucleospin Rapid lyse according to the manufacturer’s instructions. Quantification of tachyzoite numbers was done using *T*. *gondii*-specific quantitative TaqMan Real-time PCR as described earlier [36]. Assays were performed in triplicates and EC_50_ values were calculated using the logit-log algorithm and are indicated with 95% confidence intervals.

In parallel, MICs for C3- and C4-adapted and WT *T. gondii* ME49 were determined using HFF monolayers grown in 96-well plates inoculated with 10^3^ freshly purified tachyzoites by applying serial two-fold dilutions of the compounds starting at 50 μM as previously reported [44]. After five days, 96-well plates were examined under the microscope and MIC values were given as the first concentrations at which rosettes were visible [44].

### 4.5. Transmission Electron Microscopy (TEM)

TEM was conducted as described earlier [44,61]. Briefly, confluent HFF in T25 flasks were inoculated with 1 × 10^6^ *T. gondii* ME49 tachyzoites. At 24 h post-infection, C3 or C4 were added at 1 μM, whereas negative control cultures received complemented DMEM containing 0.005% DMSO (solvent control). As a positive control for visualization of drug-induced alterations, cultures were treated with 1 µM DB745 [48]. Samples were embedded at different time points (6, 12, 24 and 48 h). For fixation, specimens were washed with a sodium cacodylate solution, and a cacodylate buffer containing 2% glutaraldehyde was added. Fixed cells were then gently scraped from the flasks and were fixed for 2 h at room temperature or overnight at 4 °C. Following several washes in cacodylate buffer, specimens were post-fixed in 2% osmium tetroxide in the cacodylate buffer, followed by stepwise dehydration in a graded series (30, 50, 70, 90 and 3 × 100%) of ethanol. They were finally embedded in Epon-182 resin, and polymerization of the resin was carried out at 60 °C. Ultrathin sections (80 nm) were cut using an ultramicrotome (Reichert and Jung, Vienna, Austria) and placed onto 200 mesh formvar-carbon-coated nickel grids (Plano GmbH, Marburg, Germany) and were stained with Uranyless^®^ and lead citrate (both from Electron Microscopy Sciences, Hatfield, PA, USA). Imaging was performed on a FEI Morgagni TEM equipped with a Morada digital camera system (12 Megapixel) operating at 80 kV [44,61]. Quantification of ultrastructural alterations in the parasitophorous vacuoles and in individual tachyzoites (mitochondrial changes, vacuolization and other cytoplasmic alterations) was performed by visual electron microscopical inspection of at least 100 PVs per specimen.

### 4.6. Evaluation of the MMP by Tetramethyl Rhodamine, Ethyl Ester (TMRE) Uptake Assay

To check whether C3 and C4 could interfere with the mitochondrial membrane potential (MMP) in host cells and/or parasites, TMRE assays were conducted essentially as described. For this, 5 × 10^5^ HFF were seeded into T25 culture flasks in DMEM and maintained in culture for 72 h to reach ~80% confluency. HFF monolayers were left either non-infected or were infected with 2 × 10^5^ *T. gondii* ME49 tachyzoites. After another 48 h, the medium was removed from infected and non-infected T25 flasks and was replaced either with a medium containing 0.5 µM C3 or C4, or in the case of non-treated controls, a medium containing 0.05% DMSO. As positive controls, the two mitochondrial uncouplers carbonyl cyanide 4-(trifluoromethoxy) phenylhydrazone (FCCP) and carbonyl cyanide m-chlorophenylhydrazone (CCCP) were applied at a concentration of 80 and 50 µM, respectively. Pyrimethamine (PYR) was applied at 0.5 µM as a control drug not affecting the MMP. The infected and non-infected HFF monolayers were treated with C3, C4 or PYR for 3.5 h, and FCCP and CCCP were added during 10 min before the end of the treatment. Cultures receiving no treatment were used as positive controls (100% TMRE uptake). Following the treatments, cells were thrice washed with Hanks Balanced Salt Solution (HBSS), were incubated with TMRE (500 nM) for 30 min and were subsequently washed five times with HBSS. Finally, 3 mL of HBSS was added to each T25 flask and cells were removed with a cell scraper and were passed through a G25 needle. The resulting lysates were passed through 3.0-micron polycarbonate membranes and were distributed into 96-well plates (100 µL/well). Fluorescence was measured using a Hidex Sense microplate reader instrument (Agilent Technologies, Santa Clara, CA, USA) with the excitation wavelength 544/20 nm and emitted light collected at 590/20 nm. Values from wells belonging to the same experimental conditions were summed and the mean TMRE uptake in each condition was calculated from three biological replicates. Results are shown as the mean of TMRE uptake in each condition plus/minus standard deviation, and statistical significance was calculated using Student’s *t*-test. (*p* ≤ 0.05).

### 4.7. Drug Treatments for Extended Periods of Time and Generation of C3 and C4 Adapted T. gondii ME49 Clonal Strains

To check whether C3 and C4 exert parasiticidal or parasitostatic activity, we carried-out a long-term treatment experiment lasting over a period with a maximum of 20 days, with the addition of a fresh medium containing compounds every three days. For this, HFF-grown T25 culture flasks were infected with 2 × 10^5^ *T. gondii* ME49 tachyzoites, and treatments with 0.5 µM of either C3 or C4 were initiated at 3.5 h post-infection. Drugs were removed at different time points (days 3, 6, 9, 13, 16 and 20) and were replaced with complemented DMEM. Cultures were checked daily using light microscopy to observe and assess the progression of plaque formation. Since tachyzoites were able to recover full proliferation in the presence or absence of both drugs, we decided to generate in vitro adapted *T. gondii* clones. Thus, HFF monolayers in T25 flasks were infected with 2 × 10^5^ *T. gondii* ME49 tachyzoites and treatments (0.5 µM C3 or C4) were initiated at 3.5 h post-infection. Media containing C3 or C4 were renewed at day 3. After six days of treatment, the drug-containing medium was removed and was replaced with a complemented medium without drugs, and cultures were further maintained for four days when plaque formation became evident. On day 10, infected monolayers were washed with phosphate-buffered saline (PBS 1X), tachyzoites were collected, counted and cloned by limited dilution (0.2 tachyzoites/0.2 mL medium) in HFF monolayers grown in 96-well plates. After eight–ten days of culture at 37 °C and 5% CO_2_, wells were inspected with light microscopy and cells from those containing a single plaque (one area of lysis) were transferred into a fresh T25 flask to infect and proliferate within HFF monolayers. The resulting cultures were suspended in heat inactivated fetal calf serum containing 10% DMSO and stored at −150 °C (two cryotubes per clone). Subsequently, stabilates were reintroduced into the culture and after one passage were used for EC_50_ and MIC determination (see above). For proteomics, WT *T. gondii* ME49 and C3 and C4 clones were cultured in HFF monolayers in T75 flasks. Pellets were obtained as previously described [44] and stored at −80 °C.

### 4.8. Differential Affinity Chromatography (DAC)

Epoxy-activated Sepharose 6B (Merck, Darmstadt, Germany) matrices conjugated to either C3, C4 or tyrosine (as an internal control with similar chemical structure as C3 and C4 and ineffective against *T. gondii*) were prepared as previously described [61,62].

In addition, a mock column with resin treated in the same way but without C3, C4 or tyrosine, was prepared and was used in tandem with C3, C4 or tyrosine (mock first, then compound) [61,62]. Frozen pellets of *T. gondii* ME49 tachyzoites were resuspended in ice-cold extraction buffer containing 1% Triton X-100 and 1% HALT proteinase inhibitor cocktail (ThermoFisher Scientific, Reinach, Switzerland)), vortexed thoroughly and centrifuged at 10,000× *g* for 30 min. The resulting supernatants were subjected to affinity chromatography [61,62]. Next, epoxy-activated Sepharose 6B (Merck, Darmstadt, Germany) matrices conjugated to compound C3 or C4 were prepared as previously described [61,62], and one column was coupled to tyrosine (internal control with similar chemical structure to C3 and C4 and ineffective against *T. gondii*). Mock columns were prepared in the same way, but without compounds (without C3, C4 or tyrosine) and were used in tandem with C3, C4 or tyrosine (mock first, then compound) [61,62]. Bound proteins were eluted with 50 mM acetic acid (5 mL per column), lyophilized and stored at −80 °C for subsequent proteomic analysis (see below).

### 4.9. Proteomics

The DAC samples were resuspended in 10 µL of a solution containing 8 M urea/100 mM Tris pH 8, reduced with 0.1 M dithiothreitol (DTT) for 30 min at 37 °C, alkylated with 0.5 M iodoacetamide (IAA) (Sigma, Buchs, Switzerland) for 30 min at 37 °C and digested with LysC 2 h at 37 °C followed by Trypsin at room temperature overnight. The digests were analyzed by nano-liquid chromatography on a Dionex, Ultimate 3000 (ThermoFischer Scientific, Reinach, Switzerland) coupled to a timsTOF Pro through a CaptiveSpray source (Bruker Daltonics, Bremen, Germany) with an endplate offset of 500 V, a drying temperature of 200 °C and with the capillary voltage fixed at 1.6 kV. A volume of 2 µL from the protein digests was loaded onto a pre-column (C18 PepMap 100, 5 µm, 100 A, 300 µm i.d. × 5 mm length, ThermoFisher Scientific, Reinach, Switzerland) at a flow rate of 10 µL/min with 0.05% trifluoroacetic acid (TFA) in water/acetonitrile 98:2 (Merck, Buchs, Switzerland.) After loading, peptides were eluted in back flush mode onto a homemade C18 CSH (Waters, Baden, Switzerland) column (1.7 μm, 130 Å, 75 μm × 20 cm) by applying a 70-min gradient of 5% acetonitrile to 40% water/0.1% formic acid at a flow rate of 250 nLmin.

The TimsTOF Pro was operated in data-dependent acquisition (DDA) mode using the Parallel Acquisition SErial Fragmentation (PASEF) option. The mass range was set between 100 and 1700 m/z, with 10 PASEF scans in a mobility range between 0.7 and 1.4 V s/cm^2^. The accumulation time was set to 2 ms, and the ramp time was set to 100 ms. Fragmentation was triggered at 20,000 arbitrary units (au), and peptides (up to charge 5) were fragmented using collision induced dissociation with a spread between 20 and 59 eV.

The data was searched and quantified with fragpipe [63] version 20.0 with the following parameters: database was *Toxoplasma gondii* entries from uniprot [64] (strain ATCC 50861, release July 2023), to which common contaminants were added; precursor and fragment mass tolerances were set to ±20 ppm and ±0.05 Da, respectively; protein digestion was set to trypsin, with a maximum of three missed cleavages; variable modifications allowed were oxidation on methionine and protein N-terminal acetylation; carbamidomethylation of cysteines was given as fixed modification; the minimum number of matched fragments was set to five; validation was achieved with Percolator (MSBooster enabled); protein inference was performed with Protein Prophet and results were filtered at 1% False Discovery Rate (FDR) for protein level. Quantification was conducted without match between runs. Protein groups identified by only 1 peptide were removed from the list of positively identified proteins.

Next to fragpipe’s measure of quantification (FragI), MaxLFQ values were reported by the software. Top3 [65] values were calculated as the sum of the three most intense peptide forms for each protein, having first normalized the peptide forms by variance stabilization normalization (vsn) [66]. After filtering off contaminants, both the peptide forms and the FragI and MaxLFQ were re-normalized by vsn. In order the calculate consistent log2 fold change, missing values were replaced at the protein level by the lowest value of the corresponding sample. iBAQ values were calculated as described [67]. The empirical cumulative distribution of the log2 fold changes was obtained from the ecdf function of the R base package.

For the analysis of the adapted tachyzoites, three technical and three biological replicates were subjected to whole-cell shotgun mass spectrometry. Cell pellets were re-suspended in 8 M urea/100 mM Tris-HCl pH. Proteins were reduced and alkylated as described [68]. Samples were diluted by addition of 1/10-volume of 50 mM Tris/HCl pH 8.0 before protein precipitation with 5 volumes of cold (−20 °C) acetone (Merck, Buchs, Switzerland) over night at −20 °C. Proteins were pelleted by centrifugation at 16,000× *g* for 10 min at 4 °C and the acetone supernatant was discarded. Pellets were dried in ambient air for 15 min and stored at −20 °C until use. Proteins were re-dissolved in 8 M urea/50 mM Tris-HCl (Sigma, Buchs, Switzerland) pH 8, and protein content was determined by bicinchoninic acid (BCA, ThermoFisher Scientific, Reinach, Switzerland) assay after 1:10 (*v*/*v*) dilution with water. Urea was then diluted to 1.6 M by addition of 20 mM Tris-HCl pH 8.0/2 mM calcium dichloride before digestion of the proteins with trypsin overnight at room temperature. Digestions were stopped by adding 1/20-volume of 20% (*v*/*v*) tri-fluoroacetic acid (TFA, (Merck, Buchs, Switzerland). The digests underwent nano-liquid chromatography analysis using a Nano Elute2 connected to a timsTOF HT, via a CaptiveSpray source (Bruker Daltonics, Bremen, Germany). The analysis was conducted with an endplate offset of 500 V and a drying temperature of 200 °C with the capillary voltage fixed at 1.6 kV. For the chromatographic separation, a volume of 5 µL corresponding to 500 ng protein digest was loaded onto a pre-column (C18 PepMap 100, 5 µm, 100A, 300 µm i.d. × 5 mm length, ThermoFisher Scientific, Reinach, Switzerland) and subsequently eluted in back flush mode onto a Bruker 10 cm pulled emitter column (ionOpticks) (1.9 μm, 75 μm) by applying a 30 min gradient of 5% acetonitrile to 30% water/0.1% formic acid, at a flow rate of 500 nL/min. The timsTOF HT was operated either in DDA or data-independent acquisition (DIA) mode using the Parallel Acquisition SErial Fragmentation (PASEF) mode. For the DDA method, the mass range was set between 100 and 1700 m/z, with 8 PASEF scans between 0.75 and 1.35 V s/cm^2^. The accumulation and ramp time were set to 100 ms. Fragmentation was triggered at 15,000 arbitrary units (au), and peptides (up to charge 5) were fragmented using collision induced dissociation with a spread between 20 and 75 eV.

The dia-PASEF acquisition method was set with 47 isolation windows of 26 *m*/*z* width, including an overlap of 1 *m*/*z*. Isolation windows were associated with ion mobility at a range from 0.7 to 1.45 V s/cm^2^. TIMS accumulation and separation were both set at 100 ms.

The DDA data were used to produce a spectral library with fragpipe [63] version 20.0 with the following parameters: database was *T. gondii* ME49 from the Eukaryotic Pathogen, Vector and Host Informatics Resource [69] (release 55), to which common contaminants were added; precursor and fragment mass tolerances were set to ±20 ppm and ±0.05 Da, respectively; protein digestion was set to trypsin, with a maximum of three missed cleavages; variable modifications allowed were oxidation on methionine and protein N-terminal acetylation; carbamidomethylation of cysteines was given as fixed modification; the minimum number of matched fragments was set to 5; validation was achieved with Percolator (MSBooster enabled); filtering was conducted at 1% FDR for protein level.

DIA data was analyzed by Spectronaut version 18.1.230626.50606 (Biognosis, Schlieren, Switzerland) with factory settings and the spectral library as described above, excluding single hit proteins. Potential contaminants were then removed for further analysis. Missing Spectronaut protein intensity values were imputed in the following manner: if there was at most one detection in a replicate group, then the remaining missing values were imputed by a random draw from a Gaussian distribution of width 0.3 × sample standard deviation and shifted left from the sample mean mu by 2.5 × sample standard deviation; all other missing values were replaced by the Maximum Likelihood Estimation method [70] for differential expression tests using the moderated *t*-test [71]. For multiple test correction, the R tool fdrtool [72] was applied. Significance criteria using 20 imputation cycles were applied as described in Ref. [73] (paragraph 4.9), imposing a minimum log2 fold change of ≥ 0.58 in absolute value, and a maximum adjusted *p*-value of 0.05 (the latter value reachable at asymptotically high log2 fold changes).

For each drug, three technical and three biological replicates were subjected to whole-cell shotgun mass spectrometry.

## Figures and Tables

**Table 1:** Half maximal effective concentrations (EC_50_) of C3 and C4 on *T. gondii* tachyzoites and HFF host cells. The proliferation of *T. gondii* β-Gal was measured by quantification of β-Galactosidase activity, while *T. gondii* ME49 proliferation was measured by quantitative real-time PCR.

	*T. gondii* β-Gal		HFF
	EC_50_ When Added Concomitantly to Infection (nM)	EC_50_ When Added 3 h Post-Infection (nM)	No Viability Impairment at (µM)
Compound			
C3	28 (27–30)	23 (20–26)	25
C4	30 (40–20)	20 (15–25)	25
	*T. gondii* ME49		
C3	31 (16–62)		
C4	13 (6–27)		

**Table 2:** List of the fourteen proteins specifically binding to C3 columns as identified by DAC followed by mass spectrometry. See Supplementary Table S1 for the full dataset. The relative abundances (rAbu) based on iBAQ sum up to a total of 1,000,000 for each sample. The proteins are listed according to their decreasing rAbu values.

ToxoDB ORF	Annotation	rAbu C3
TGME49_262690	ribosomal protein RPL27	741
TGME49_244110	nucleosome assembly protein (nap) protein	324
TGME49_309740	LSM domain-containing protein	100
TGME49_321520	hypothetical protein	91
TGME49_279390	proliferation-associated protein 2G4, putative	80
TGME49_319870	ubiquitin-conjugating enzyme subfamily protein	77
TGME49_313560	60S ribosomal protein L7a, putative	71
TGME49_248810	nuclear factor NF7	54
TGME49_231850	serine-threonine phosophatase 2C (PP2C)	51
TGME49_209170	hypothetical protein	43
TGME49_272400	casein kinase ii regulatory subunit protein	43
TGME49_227810	rhoptry kinase family protein ROP11 (incomplete catalytic triad)	35
TGME49_256050	signal recognition particle 14kd protein	28
TGME49_305340	corepressor complex CRC230	9

**Table 3:** List of the twenty most abundant proteins binding to C4 columns as identified by DAC followed by mass spectrometry. See Supplementary Table S1 for the full dataset. The relative abundances (rAbu) based on iBAQ sum up to a total of 1,000,000 for each sample. The proteins are listed according to their decreasing rAbu values.

ToxoDB ORF	Annotation	rAbu C4
TGME49_213280	SAG-related sequence SRS25	22,200
TGME49_253690	hypothetical protein (putative transmembrane protein)	15,804
TGME49_316710	hypothetical protein	4878
TGME49_266060	ribosomal protein RPSA	3977
TGME49_288245	hypothetical protein	2980
TGME49_231160	hypothetical protein	2724
TGME49_226570	hypothetical protein	1943
TGME49_285870	SAG-related sequence SRS20A	1652
TGME49_319560	microneme protein MIC3	1600
TGME49_289680	Ras-related protein Rab11	1461
TGME49_263630	hypothetical protein	1426
TGME49_214410	hypothetical protein	1316
TGME49_214770	small GTP binding protein rab1a, putative	1305
TGME49_225555	hypothetical protein	1284
TGME49_308020	SAG-related sequence SRS57	1218
TGME49_249900	adenine nucleotide translocator, putative	1171
TGME49_233450	SAG-related sequence SRS29A	1156
TGME49_308840	SAG-related sequence SRS51	1153
TGME49_245490	microneme protein MIC8	1126
TGME49_227920	hypothetical protein	1043

**Table 4:** List of the twenty most abundant common proteins binding to both C3- and C4-affinity columns as identified by DAC followed by mass spectrometry. See Supplementary Table S1 for the full dataset. The relative abundances (rAbu) based on iBAQ sum up to a total of 1,000,000 for each sample. The proteins are listed according to their decreasing rAbu values in the C3 dataset.

ToxoDB ORF	Annotation	rAbu C3	rAbu C4
TGME49_309810	ribosomal protein RPP2	3431	3473
TGME49_249250	ribosomal protein RPL35A	3406	214
TGME49_262670	ribosomal protein RPL18A	2651	522
TGME49_313390	ribosomal protein RPL6	2390	482
TGME49_261570	ribosomal protein RPL7A	2348	372
TGME49_205340	ribosomal protein RPS12	2026	493
TGME49_292130	ribosomal protein RPL13A	1678	447
TGME49_238070	glutaredoxin domain-containing protein	1666	332
TGME49_267060	ribosomal protein RPL14	1540	317
TGME49_320050	ribosomal protein RPL5	1492	223
TGME49_204020	ribosomal protein RPL8	1487	114
TGME49_238250	ribosomal protein RPL36	1422	517
TGME49_245680	ribosomal protein RPL21	1362	219
TGME49_260260	ribosomal protein RPP1	1354	2505
TGME49_314810	ribosomal protein RPL7	1325	666
TGME49_270380	ribosomal protein RPS13	1265	1846
TGME49_210690	ribosomal protein RPS6	1242	759
TGME49_215470	ribosomal protein RPL10A	1090	400
TGME49_218820	alba 2	1046	753
TGME49_232230	ribosomal protein RPL30	986	50

**Table 5:** Half maximal effective concentrations (EC_50_) and minimal inhibitory concentration (MIC) of the gold (III) complex C3 and its C4 ligand against *T. gondii* ME49 wild type tachyzoites, and three cloned strains, each derived from C3-adapted or C4-adapted *T. gondii*. EC_50_ values were determined by quantitative real-time PCR and are given at 95% confidence interval (CI); LI is the inferior limit of CI and LS is the superior limit of CI. The corresponding dose–response curves are shown in Supplementary Figure S3.

	EC_50_ (nM)	MIC (µM)
C3		
*T. gondii* Me49 wildtype (WT)	31 (16–62)	25
P1F3	45 (28–70)	25
P1E5	90 (54–150)	25
P2F5	51 (31–84)	25
**C4**		
*T. gondii* Me49 wildtype (WT)	13 (6–27)	25
P2F3	11 (6–18)	25
P1G10	10 (4–23)	25
P3E2	6 (4–9)	25

**Table 6:** List of twenty differentially expressed proteins in clones adapted to compound C3 as compared to *T. gondii* ME49 WT strain, for upregulated (FC ≥ 1.5; *p* ≤ 0.05) and for downregulated (FC ≤ 0.66; *p* ≤ 0.05). Whole cell shotgun MS was performed on three clones per drug and three replicates per clone.

ToxoDB ORF	Product Description	C3_Clone	Fold Change (FC)
Upregulated ↑			
TGME49_258700	transporter, major facilitator family protein	P1F3	6.5
TGME49_225480	hypothetical protein	P1E5	6.4
TGME49_248140	hypothetical protein	P1E5	5.6
TGME49_262120	IQ calmodulin-binding motif domain-containing protein	P1E5	3.3
TGME49_253360	SNARE protein STX20	P1E5	3.2
TGME49_306300	hypothetical protein	P1E5	3.2
TGME49_257160	hypothetical protein	P1E5	3.1
TGME49_261740	rhoptry protein ROP47	P1E5	2.8
TGME49_293480	MoeA N-terminal region (domain I and II) domain-containing protein	P1F3	2.6
TGME49_250950	KRUF family protein	P1E5	2.5
TGME49_203630	ribosomal protein RPL44	P1E5	2.4
TGME49_315290	hypothetical protein	P1E5	2.4
TGME49_214410	hypothetical protein	P1E5	2.3
TGME49_268730	glutaredoxin 1	P1E5	2.3
TGME49_268740	hypothetical protein	P1E5	2.2
TGME49_234180	hypothetical protein	P1E5	2.1
TGME49_238200	alpha/beta hydrolase fold domain-containing protein	P2F5/P1E5	2/1.6
TGME49_219690	hypothetical protein	P1E5	2.0
TGME49_202940	hypothetical protein	P1E5	2.0
TGME49_251180	KRUF family protein	P1E5/P1F3/P2F5	2/1.7/1.5
Downregulated ↓			
TGME49_248200	ribosomal RNA (adenine(1779)-N(6)/adenine(1780)-N(6))-dimethyltransferase, putative	P1E5	0.1
TGME49_209755	cyst matrix protein MAG2	P1E5	0.1
TGME49_253350	hypothetical protein	P1E5	0.3
TGME49_297110	kinesin motor domain-containing protein	P1E5	0.3
TGME49_273780	SWI2/SNF2-containing protein	P1E5	0.4
TGME49_280570	SAG-related sequence SRS35A	P1E5	0.4
TGME49_272900	DNA repair protein RAD51	P1E5	0.4
TGME49_281900	SET domain containing lysine methyltransferase KMTox	P1E5	0.4
TGME49_247390	ATPase, AAA family protein	P1E5	0.4
TGME49_280390	HEAT repeat-containing protein	P1E5	0.5
TGME49_320190	SAG-related sequence SRS16B	P1E5	0.5
TGME49_318470	AP2 domain transcription factor AP2IV-4	P1E5	0.5
TGME49_297870	inner membrane complex protein IMC36	P1E5	0.5
TGME49_214970	DNA replication licensing factor, putative	P1E5	0.5
TGME49_301170	SAG-related sequence SRS19D	P1E5	0.5
TGME49_244500	Tubulin-tyrosine ligase family protein	P1E5	0.5
TGME49_290970	serine palmitoyltransferase SPT2	P1E5	0.5
TGME49_219700	DNA replication licensing factor MCM4, putative	P1E5	0.5
TGME49_237220	DNA replication licensing factor Mcm7, putative	P1E5	0.5
TGME49_262990	hypothetical protein	P1E5	0.5

**Table 7:** List of twenty most upregulated proteins and six downregulated proteins in clones adapted to compound C4 as compared to *T. gondii* ME49 WT strain, for upregulated (FC ≥ 1.5; *p* ≤ 0.05) and for downregulated (FC ≤ 0.66; *p* ≤ 0.05). Whole cell shotgun MS was performed on three clones per drug and three replicates per clone.

ToxoDB ORF	Product Description	C4_Clone	Fold Change (FC)
Upregulated ↑			
TGME49_306300	hypothetical protein	P1G10	7.2
TGME49_258700	transporter, major facilitator family protein	P2F3	4.2
TGME49_248140	hypothetical protein (putative transmembrane protein)	P3E2	4.1
TGME49_234980	hypothetical protein (putative transmembrane protein)	P2F3	3.8
TGME49_277740	zinc finger, C3HC4 type (RING finger) domain-containing protein	P2F3	2.8
TGME49_268860	enolase 1	P2F3	2.8
TGME49_265190	Ulp1 protease family, C-terminal catalytic domain-containing protein	P2F3	2.7
TGME49_276120	histone lysine methyltransferase, SET, putative	P2F3	2.3
TGME49_220170	hypothetical protein	P2F3	2.2
TGME49_200440	hypothetical protein	P2F3	2.2
TGME49_273060	ribosomal protein S17, putative	P2F3	2.1
TGME49_273550	hypothetical protein	P2F3	2.1
TGME49_293480	MoeA N-terminal region (domain I and II) domain-containing protein	P3E2	2
TGME49_238200	alpha/beta hydrolase fold domain-containing protein	P2F3/P1G10	2.01/1.9
TGME49_284580	ribose-phosphate diphosphokinase subfamily protein	P2F3	2
TGME49_211470	Fcf2 pre-rRNA processing protein	P2F3	2
TGME49_229260	basal complex component BCC4	P2F3	2
TGME49_226640	zinc binding protein, putative	P2F3	2
TGME49_224932	hypothetical protein	P2F3	1.9
TGME49_297220	AMP-binding enzyme domain-containing protein	P2F3	1.9
Downregulated ↓			
TGME49_309580	transporter, major facilitator family protein	P2F3	0.1
TGME49_268220	hypothetical protein	P2F3	0.2
TGME49_305870	DAD family protein	P1G10	0.4
TGME49_305610	hypothetical protein	P2F3	0.5
TGME49_229140	MaoC family domain-containing protein	P1G10	0.6
TGME49_320588	glycosyl hydrolases family 35 protein	P2F3	0.6

**Table 8:** List of differentially expressed proteins in C3- and C4-recovered clones as compared to *T. gondii* ME49 WT tachyzoites. Eleven proteins were upregulated (FC ≥ 1.5; *p* ≤ 0.05), one was downregulated (FC ≤ 0.66; *p* ≤ 0.05) in both C3 and C4 clones, and three proteins had a divergent expression between C3 and C4 clones.

	Annotation	C3_Clone	FC	C4_Clone	FC
Upregulated C3↑ C4 ↑					
TGME49_306300	hypothetical protein	P1E5	3.2	P1G10	7.2
TGME49_258700	transporter, major facilitator family protein	P1F3	6.5	P2F3	4.2
TGME49_248140	hypothetical protein (putative transmembrane protein)	P1E5	5.6	P3E2	4.1
TGME49_293480	MoeA N-terminal region (domain I and II) domain-containing protein	P1F3	2.6	P3E2	2.0
TGME49_238200	alpha/beta hydrolase fold domain-containing protein	P1E5/P2F5	1.62/2.03	P2F3/P1G10	2.0/1.9
TGME49_211470	Fcf2 pre-rRNA processing protein	P2F5	1.8	P2F3	2.0
TGME49_297220	AMP-binding enzyme domain-containing protein	P1E5	2.0	P2F3	1.9
TGME49_277920	hypothetical protein (putative transmembrane protein)	P1E5	2.0	P2F3	1.5
TGME49_224932	hypothetical protein (putative jmjC domain protein)	P1E5	1.6	P2F3	1.9
TGME49_269660	TFIIH basal transcription factor complex helicase XPB subunit	P1E5	1.6	P2F3	1.5
TGME49_274100	hypothetical protein	P2F5	1.5	P2F3	1.6
Downregulated C3↓ C4 ↓					
TGME49_229140	MaoC family domain-containing protein	P1F3	0.5	P1G10	0.6
Divergent C3↓ C4 ↑					
TGME49_312500	hypothetical protein	P1E5	0.6	P2F3	1.5
TGME49_315590	macro domain-containing protein	P1E5	0.6	P2F3	1.9
TGME49_242720	aspartyl protease ASP5	P1E5	0.7	P2F3	1.5

## Data Availability

All data is provided in the manuscript and the Supplementary Files.

## Supplementary Materials

The following supporting information can be downloaded at: https://www.mdpi.com/article/10.3390/ijms25169067/s1.

## References

[1] Almeria S., Dubey J.P. (2021). Foodborne Transmission of Toxoplasma Gondii Infection in the Last Decade. An Overview. Res. Vet. Sci..

[2] Attias M., Teixeira D.E., Benchimol M., Vommaro R.C., Crepaldi P.H., De Souza W. (2020). The Life-Cycle of Toxoplasma Gondii Reviewed Using Animations. Parasit. Vectors.

[3] Lindsay D.S., Dubey J.P. (2020). Neosporosis, Toxoplasmosis, and Sarcocystosis in Ruminants: An Update. Vet. Clin. N. Am.-Food Anim. Pract..

[4] Dubey J.P., Murata F.H.A., Cerqueira-Cézar C.K., Kwok O.C.H., Villena I. (2021). Congenital Toxoplasmosis in Humans: An Update of Worldwide Rate of Congenital Infections. Parasitology.

[5] Dubey J.P. (2010). Toxoplasmosis of Animals and Humans.

[6] Di Cristina M., Marocco D., Galizi R., Proietti C., Spaccapelo R., Crisanti A. (2008). Temporal and Spatial Distribution of Toxoplasma Gondii Differentiation into Bradyzoites and Tissue Cyst Formation in Vivo. Infect. Immun..

[7] Konstantinovic N., Guegan H., Stäjner T., Belaz S., Robert-Gangneux F. (2019). Treatment of Toxoplasmosis: Current Options and Future Perspectives. Food Waterborne Parasitol..

[8] Montazeri M., Mehrzadi S., Sharif M., Sarvi S., Shahdin S., Daryani A. (2018). Activities of Anti-Toxoplasma Drugs and Compounds against Tissue Cysts in the Last Three Decades (1987 to 2017), a Systematic Review. Parasitol. Res..

[9] Murata Y., Sugi T., Weiss L.M., Kato K. (2017). Identification of Compounds That Suppress Toxoplasma Gondii Tachyzoites & Bradyzoites. PLoS ONE.

[10] Heloisa B., Dinorah G. (2004). The Wide Pharmacological Versatility of Semicarbazones, Thiosemicarbazones and Their Metal Complexes. Mini-Rev. Med. Chem..

[11] Khan T., Raza S., Lawrence A.J. (2022). Medicinal Utility of Thiosemicarbazones with Special Reference to Mixed Ligand and Mixed Metal Complexes: A Review. Russ. J. Coord. Chem./Koord. Khimiya.

[12] Pelosi G. (2010). Thiosemicarbazone Metal Complexes: From Structure to Activity. Open Crystallogr. J..

[13] Scaccaglia M., Rega M., Vescovi M., Pinelli S., Tegoni M., Bacci C., Pelosi G., Bisceglie F. (2022). Gallium(III)-Pyridoxal Thiosemicarbazone Derivatives as Nontoxic Agents against Gram-Negative Bacteria. Metallomics.

[14] Krchniakova M., Paukovcekova S., Chlapek P., Neradil J., Skoda J., Veselska R. (2022). Thiosemicarbazones and Selected Tyrosine Kinase Inhibitors Synergize in Pediatric Solid Tumors: NDRG1 Upregulation and Impaired Prosurvival Signaling in Neuroblastoma Cells. Front. Pharmacol..

[15] Kolesar J.M., Schelman W.R., Geiger P.G., Holen K.D., Traynor A.M., Alberti D.B., Thomas J.P., Chitambar C.R., Wilding G., Antholine W.E. (2008). Electron Paramagnetic Resonance Study of Peripheral Blood Mononuclear Cells from Patients with Refractory Solid Tumors Treated with Triapine^®^. J. Inorg. Biochem..

[16] Ansari M., Montazeri M., Daryani A., Farshadfar K., Emami S. (2020). Synthesis and in Vitro Anti-Toxoplasma Gondii Activity of a New Series of Aryloxyacetophenone Thiosemicarbazones. Mol. Divers..

[17] Bekier A., Węglińska L., Paneth A., Paneth P., Dzitko K. (2021). 4-Arylthiosemicarbazide Derivatives as a New Class of Tyrosinase Inhibitors and Anti-Toxoplasma Gondii Agents. J. Enzym. Inhib. Med. Chem..

[18] De Aquino T.M., Liesen A.P., da Silva R.E.A., Lima V.T., Carvalho C.S., de Faria A.R., de Araújo J.M., de Lima J.G., Alves A.J., de Melo E.J.T. (2008). Synthesis, Anti-Toxoplasma Gondii and Antimicrobial Activities of Benzaldehyde 4-Phenyl-3-Thiosemicarbazones and 2-[(Phenylmethylene)Hydrazono]-4-Oxo-3-Phenyl-5-Thiazolidineacetic Acids. Bioorg. Med. Chem..

[19] Dzitko K., Paneth A., Plech T., Pawełczyk J., Stączek P., Stefańska J., Paneth P. (2014). 1,4-Disubstituted Thiosemicarbazide Derivatives Are Potent Inhibitors of Toxoplasma Gondii Proliferation. Molecules.

[20] Gomes M.A.G.B., Carvalho L.P., Rocha B.S., Oliveira R.R., De Melo E.J.T., Maria E.J. (2013). Evaluating Anti-Toxoplasma Gondii Activity of New Serie of Phenylsemicarbazone and Phenylthiosemicarbazones in Vitro. Med. Chem. Res..

[21] Paneth A., Weglinska L., Bekier A., Stefaniszyn E., Wujec M., Trotsko N., Dzitko K. (2019). Systematic Identification of Thiosemicarbazides for Inhibition of Toxoplasma Gondii Growth In Vitro. Molecules.

[22] Tenório R.P., Carvalho C.S., Pessanha C.S., De Lima J.G., De Faria A.R., Alves A.J., De Melo E.J.T., Góes A.J.S. (2005). Synthesis of Thiosemicarbazone and 4-Thiazolidinone Derivatives and Their in Vitro Anti-Toxoplasma Gondii Activity. Bioorg. Med. Chem. Lett..

[23] Faa G., Gerosa C., Fanni D., Lachowicz J.I., Nurchi V.M. (2017). Gold–Old Drug with New Potentials. Curr. Med. Chem..

[24] Liu Y., Lu Y., Xu Z., Ma X., Chen X., Liu W. (2022). Repurposing of the Gold Drug Auranofin and a Review of Its Derivatives as Antibacterial Therapeutics. Drug Discov. Today.

[25] Andrade R.M., Chaparro J.D., Capparelli E., Reed S.L. (2014). Auranofin Is Highly Efficacious against Toxoplasma Gondii In Vitro and in an In Vivo Experimental Model of Acute Toxoplasmosis. PLoS Negl. Trop. Dis..

[26] Almeida C.M., Pedro P.H., Nascimento É.C.M., Martins J.B.L., Chagas M.A.S., Fujimori M., De Marchi P.G.F., França E.L., Honorio-França A.C., Gatto C.C. (2022). Organometallic Gold (III) and Platinum (II) Complexes with Thiosemicarbazone: Structural Behavior, Anticancer Activity, and Molecular Docking. Appl. Organomet. Chem..

[27] Dominelli B., Correia J.D.G., Kühn F.E. (2018). Medicinal Applications of Gold(I/III)-Based Complexes Bearing N-Heterocyclic Carbene and Phosphine Ligands. J. Organomet. Chem..

[28] Dou Z., Carruthers V.B. (2011). Cathepsin Proteases in Toxoplasma Gondii. Adv. Exp. Med. Biol..

[29] Xue J., Jiang W., Chen Y., Gong F., Wang M., Zeng P., Xia C., Wang Q., Huang K. (2017). Thioredoxin Reductase from Toxoplasma Gondii: An Essential Virulence Effector with Antioxidant Function. FASEB J..

[30] Radisavljević S., Petrović B. (2020). Gold(III) Complexes: An Overview on Their Kinetics, Interactions With DNA/BSA, Cytotoxic Activity, and Computational Calculations. Front. Chem..

[31] Linciano P., Moraes C.B., Alcantara L.M., Franco C.H., Pascoalino B., Freitas-Junior L.H., Macedo S., Santarem N., Cordeiro-da-Silva A., Gul S. (2018). Aryl Thiosemicarbazones for the Treatment of Trypanosomatidic Infections. Eur. J. Med. Chem..

[32] Houngue H.D., Aguida B.S., Kassehin U.C., Poupaert J.H., Gbaguidi F.A., Codjo H. (2017). Biological Evaluation of a Series of Thiosemicarbazones Targeting the Large Subunit Ribosomal Protein EL42 from Human 80S Ribosomes. MOJ Bioorganic Org. Chem..

[33] Hofflin J.M., Remington J.S. (1987). Clindamycin in a Murine Model of Toxoplasmic Encephalitis. Antimicrob. Agents Chemother..

[34] Chew W.K., Segarra I., Ambu S., Mak J.W. (2012). Significant Reduction of Brain Cysts Caused by Toxoplasma Gondii after Treatment with Spiramycin Coadministered with Metronidazole in a Mouse Model of Chronic Toxoplasmosis. Antimicrob. Agents Chemother..

[35] Müller J., Anghel N., Imhof D., Hänggeli K., Uldry A.C., Braga-Lagache S., Heller M., Ojo K.K., Ortega-Mora L.M., Van Voorhis W.C. (2022). Common Molecular Targets of a Quinolone Based Bumped Kinase Inhibitor in Neospora Caninum and Danio Rerio. Int. J. Mol. Sci..

[36] Hänggeli K.P.A., Hemphill A., Müller N., Heller M., Uldry A.C., Braga-Lagache S., Müller J., Boubaker G. (2023). Comparative Proteomic Analysis of Toxoplasma Gondii RH Wild-Type and Four SRS29B (SAG1) Knock-Out Clones Reveals Significant Differences between Individual Strains. Int. J. Mol. Sci..

[37] Gc K., To D., Jayalath K., Abeysirigunawardena S. (2019). Discovery of a Novel Small Molecular Peptide That Disrupts Helix 34 of Bacterial Ribosomal RNA. RSC Adv..

[38] Cao S., Yang J., Fu J., Chen H., Jia H. (2021). The Dissection of SNAREs Reveals Key Factors for Vesicular Trafficking to the Endosome-like Compartment and Apicoplast via the Secretory System in Toxoplasma Gondii. mBio.

[39] Fu J., Zhao L., Yang J., Chen H., Cao S., Jia H. (2023). An Unconventional SNARE Complex Mediates Exocytosis at the Plasma Membrane and Vesicular Fusion at the Apical Annuli in Toxoplasma Gondii. PLoS Pathog..

[40] Fernández L., Hancock R.E.W. (2012). Adaptive and Mutational Resistance: Role of Porins and Efflux Pumps in Drug Resistance. Clin. Microbiol. Rev..

[41] Ayaz M., Subhan F., Sadiq A., Ullah F., Ahmed J., Sewell R.D. (2017). Cellular Efflux Transporters and the Potential Role of Natural Products in Combating Efflux Mediated Drug Resistance. Front. Biosci. Landmark.

[42] Jiao L., Liu Y., Yu X.Y., Pan X., Zhang Y., Tu J., Song Y.H., Li Y. (2023). Ribosome Biogenesis in Disease: New Players and Therapeutic Targets. Signal Transduct. Target. Ther..

[43] Rudra P., Hurst-Hess K., Lappierre P., Ghosha P. (2018). High Levels of Intrinsic Tetracycline Resistance in Mycobacterium Abscessus Are Conferred by a Tetracycline-Modifying Monooxygenase. Antimicrob. Agents Chemother..

[44] Müller J., Schlange C., Heller M., Uldry A.C., Braga-Lagache S., Haynes R.K., Hemphill A. (2023). Proteomic Characterization of Toxoplasma Gondii ME49 Derived Strains Resistant to the Artemisinin Derivatives Artemiside and Artemisone Implies Potential Mode of Action Independent of ROS Formation. Int. J. Parasitol. Drugs Drug Resist..

[45] Ramseier J., Imhof D., Anghel N., Hänggeli K., Beteck R.M., Balmer V., Ortega-mora L.M., Sanchez-sanchez R., Ferre I., Haynes R.K. (2021). Assessment of the Activity of Decoquinate and Its Quinoline–O–carbamate Derivatives against Toxoplasma Gondii in Vitro and in Pregnant Mice Infected with t. Gondii Oocysts. Molecules.

[46] Imhof D., Anghel N., Winzer P., Balmer V., Ramseier J., Hänggeli K., Choi R., Hulverson M.A., Whitman G.R., Arnold S.L.M. (2021). In Vitro Activity, Safety and in Vivo Efficacy of the Novel Bumped Kinase Inhibitor BKI-1748 in Non-Pregnant and Pregnant Mice Experimentally Infected with Neospora Caninum Tachyzoites and Toxoplasma Gondii Oocysts. Int. J. Parasitol. Drugs Drug Resist..

[47] Barna F., Debache K., Vock C.A., Küster T., Hemphill A. (2013). In Vitro Effects of Novel Ruthenium Complexes in Neospora Caninum and Toxoplasma Gondii Tachyzoites. Antimicrob. Agents Chemother..

[48] Kropf C., Debache K., Rampa C., Barna F., Schorer M., Stephens C.E., Ismail M.A., Boykin D.W., Hemphill A. (2012). The Adaptive Potential of a Survival Artist: Characterization of the in Vitro Interactions of Toxoplasma Gondii Tachyzoites with Di-Cationic Compounds in Human Fibroblast Cell Cultures. Parasitology.

[49] Anghel N., Balmer V., Müller J., Winzer P., Aguado-Martinez A., Roozbehani M., Pou S., Nilsen A., Riscoe M., Doggett J.S. (2018). Endochin-like Quinolones Exhibit Promising Efficacy against Neospora Caninum in Vitro and in Experimentally Infected Pregnant Mice. Front. Vet. Sci..

[50] Aspinall T.V., Joynson D.H.M., Guy E., Hyde J.E., Sims P.F.G. (2002). The Molecular Basis of Sulfonamide Resistance in Toxoplasma Gondii and Implications for the Clinical Management of Toxoplasmosis. J. Infect. Dis..

[51] Doliwa C., Escotte-Binet S., Aubert D., Velard F., Schmid A., Geers R., Villena I. (2013). Induction of Sulfadiazine Resistance in Vitro in Toxoplasma Gondii. Exp. Parasitol..

[52] Leggett H.C., Benmayor R., Hodgson D.J., Buckling A. (2013). Experimental Evolution of Adaptive Phenotypic Plasticity in a Parasite. Curr. Biol..

[53] Dixon S.E., Stilger K.L., Elias E.V., Naguleswaran A., Sullivan W.J. (2010). A Decade of Epigenetic Research in Toxoplasma Gondii. Mol. Biochem. Parasitol..

[54] Radke J.B., Worth D., Hong D., Huang S., Sullivan W.J., Wilson E.H., White M.W. (2018). Transcriptional Repression by ApiAP2 Factors Is Central to Chronic Toxoplasmosis. PLoS Pathog..

[55] Müller J., Braga S., Heller M., Müller N. (2019). Resistance Formation to Nitro Drugs in Giardia Lamblia: No Common Markers Identified by Comparative Proteomics. Int. J. Parasitol. Drugs Drug Resist..

[56] Barbasz A., Oćwieja M. (2016). Gold Nanoparticles and Ions—Friends or Foes? As They Are Seen by Human Cells U-937 and HL-60. J. Exp. Nanosci..

[57] Scaccaglia M., Pinelli S., Manini L., Ghezzi B., Nicastro M., Heinrich J., Kulak N., Mozzoni P., Pelosi G., Bisceglie F. (2024). Gold(III) Complexes with Thiosemicarbazone Ligands: Insights into Their Cytotoxic Effects on Lung Cancer Cells. J. Inorg. Biochem..

[58] Theisen T.C., Boothroyd J.C. (2022). Transcriptional Signatures of Clonally Derived Toxoplasma Tachyzoites Reveal Novel Insights into the Expression of a Family of Surface Proteins. PLoS ONE.

[59] Winzer P., Müller J., Aguado-Martínez A., Rahman M., Balmer V., Manser V., Ortega-Mora L.M., Ojo K.K., Fan E., Maly D.J. (2015). In Vitro and in Vivo Effects of the Bumped Kinase Inhibitor 1294 in the Related Cyst-Forming Apicomplexans Toxoplasma Gondii and Neospora Caninum. Antimicrob. Agents Chemother..

[60] Păunescu E., Boubaker G., Desiatkina O., Anghel N., Amdouni Y., Hemphill A., Furrer J. (2021). The Quest of the Best—A SAR Study of Trithiolato-Bridged Dinuclear Ruthenium(II)-Arene Compounds Presenting Antiparasitic Properties. Eur. J. Med. Chem..

[61] Anghel N., Müller J., Serricchio M., Jelk J., Bütikofer P., Boubaker G., Imhof D., Ramseier J., Desiatkina O., Păunescu E. (2021). Cellular and Molecular Targets of Nucleotide-Tagged Trithiolato-Bridged Arene Ruthenium Complexes in the Protozoan Parasites Toxoplasma Gondii and Trypanosoma Brucei. Int. J. Mol. Sci..

[62] Müller J., Boubaker G., Imhof D., Hänggeli K., Haudenschild N., Uldry A.C., Braga-Lagache S., Heller M., Ortega-Mora L.M., Hemphill A. (2022). Differential Affinity Chromatography Coupled to Mass Spectrometry: A Suitable Tool to Identify Common Binding Proteins of a Broad-Range Antimicrobial Peptide Derived from Leucinostatin. Biomedicines.

[63] Yu F., Haynes S.E., Teo G.C., Avtonomov D.M., Polasky D.A., Nesvizhskii A.I. (2020). Fast Quantitative Analysis of TimsTOF PASEF Data with MSFragger and IonQuant. Mol. Cell. Proteom..

[64] Bateman A. (2019). UniProt: A Worldwide Hub of Protein Knowledge. Nucleic Acids Res..

[65] Silva J.C., Gorenstein M.V., Li G.Z., Vissers J.P.C., Geromanos S.J. (2006). Absolute Quantification of Proteins by LCMSE: A Virtue of Parallel MS Acquisition. Mol. Cell. Proteom..

[66] Huber W., Von Heydebreck A., Sültmann H., Poustka A., Vingron M. (2002). Variance Stabilization Applied to Microarray Data Calibration and to the Quantification of Differential Expression. Bioinformatics.

[67] Schwanhüusser B., Busse D., Li N., Dittmar G., Schuchhardt J., Wolf J., Chen W., Selbach M. (2011). Global Quantification of Mammalian Gene Expression Control. Nature.

[68] Braga-Lagache S., Buchs N., Iacovache M.I., Zuber B., Jackson C.B., Heller M. (2016). Robust Label-Free, Quantitative Profiling of Circulating Plasma Microparticle (MP) Associated Proteins. Mol. Cell. Proteom..

[69] Alvarez-Jarreta J., Amos B., Aurrecoechea C., Bah S., Barba M., Barreto A., Basenko E.Y., Belnap R., Blevins A., Böhme U. (2024). VEuPathDB: The Eukaryotic Pathogen, Vectorãnd Host Bioinf Ormatics Resour Ce Cent Er in 2023. Nucleic Acids Res..

[70] Silver J.D., Ritchie M.E., Smyth G.K. (2009). Microarray Background Correction: Maximum Likelihood Estimation for the Normal-Exponential Convolution. Biostatistics.

[71] Kammers K., Cole R.N., Tiengwe C., Ruczinski I. (2015). Detecting Significant Changes in Protein Abundance. EuPA Open Proteom..

[72] Strimmer K. (2008). A Unified Approach to False Discovery Rate Estimation. BMC Bioinform..

[73] Uldry A.C., Maciel-Dominguez A., Jornod M., Buchs N., Braga-Lagache S., Brodard J., Jankovic J., Bonadies N., Heller M. (2022). Effect of Sample Transportation on the Proteome of Human Circulating Blood Extracellular Vesicles. Int. J. Mol. Sci..

