# Supplementary material for: Transient Adaptation of Toxoplasma gondii to Exposure by Thiosemicarbazone Drugs That Target Ribosomal Proteins Is Associated with the Upregulated Expression of Tachyzoite Transmembrane Proteins and Transporters
Source: Int J Mol Sci. 2024 Aug 21;25(16):9067. doi: 10.3390/ijms25169067 (PMC11354806; doi:10.3390/ijms25169067)

Supplementary Figures

Supplementary Figure S1. *In vitro* toxicity of compounds C1 (A) and C2 (B) against *T. gondii*-β-Gal and non-infected HFF (human foreskin fibroblast) host cells. Results are presented in % proliferation in relation to untreated control cultures (CTR). The mean values ± SE are indicated for sextuplicates.

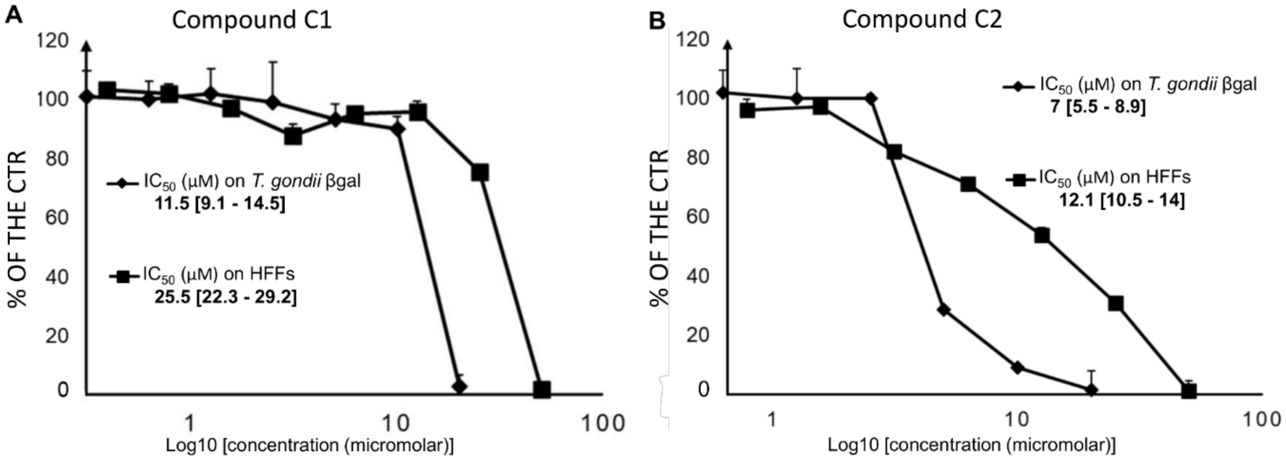

Supplementary Figure S2. Tetramethylrhodamine ethyl ester (TMRE) uptake assay showing no evidence of mitochondrial targeting by C3 and C4. TMRE uptake was measured in the presence or absence of either uncouplers (FCCP and CCCP), the standard drug pyrimethamine (PYR) and C3 and C4. Assays were carried out in T25 tissue culture flasks, and the bars represent the mean of TMRE fluorescence. Standard deviations (SD) are calculated from three biological replicates. 100% of TMRE uptake was set for the control cells in absence of uncouplers or treatments, and the corresponding percentage of TMRE fluorescence intensity is displayed on the top of each bar. Note the clear effect of FCCP and CCP, while PYR, C3 and C4 did not affect the MMP.

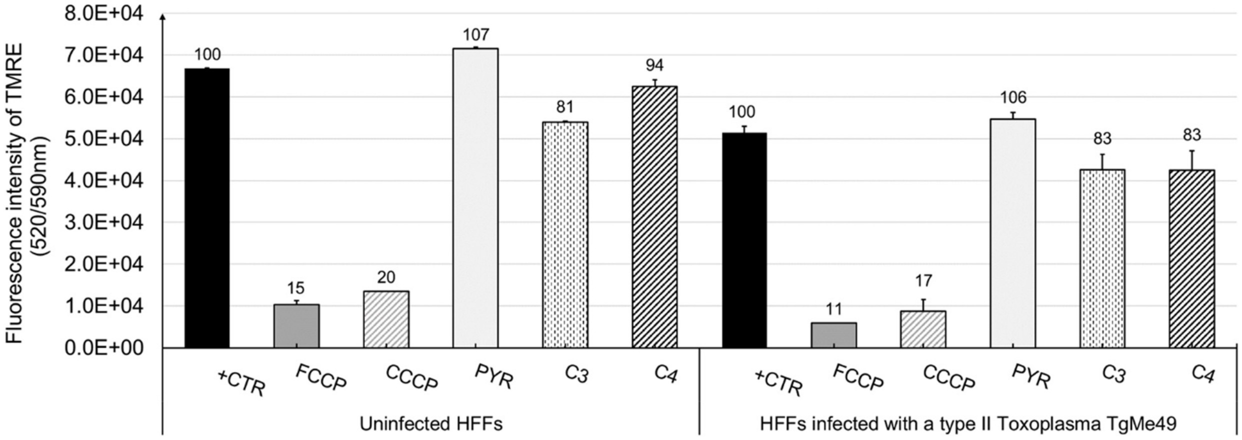

Supplementary Figure S3. Dose-response curves for the compounds C3 (A) and C4 (B) against *Toxoplasma gondii* ME49 wild-type (WT) and their respective adapted clones (3 per drug) as determined by quantitative PCR. The mean values ± SE are indicated for triplicates.

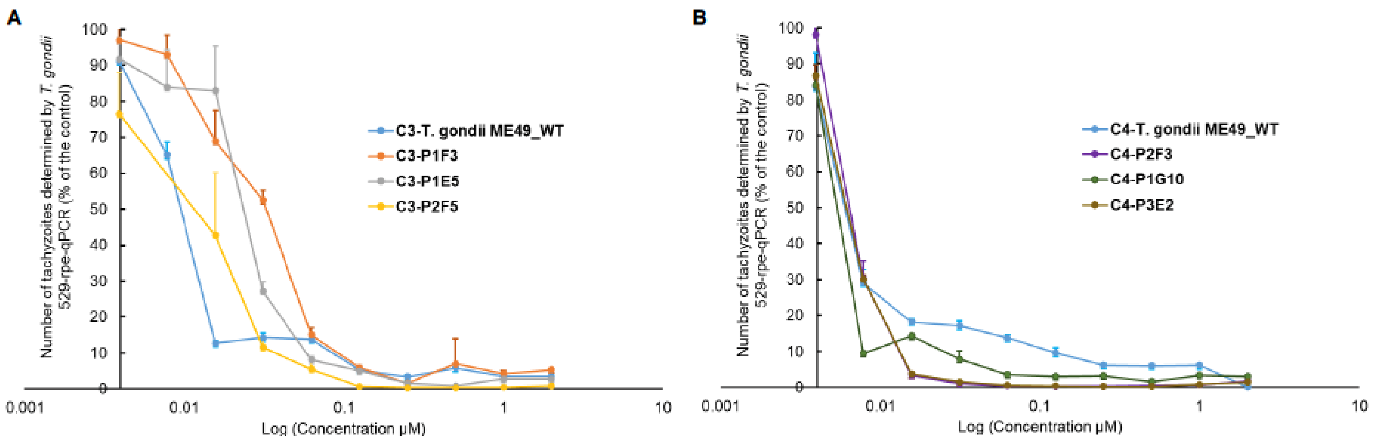

Supplementary Figure S4. Protein-protein interaction network of proteins differentially upregulated (128 proteins) in C3- and C4-clones compared to *T. gondii* wildtype tachyzoites. A small cluster of ribosomal proteins was identified. The interaction network was created by the STRING knowledgebase and software tool from the Swiss Institute of Bioinformatics (www.expasy.org).

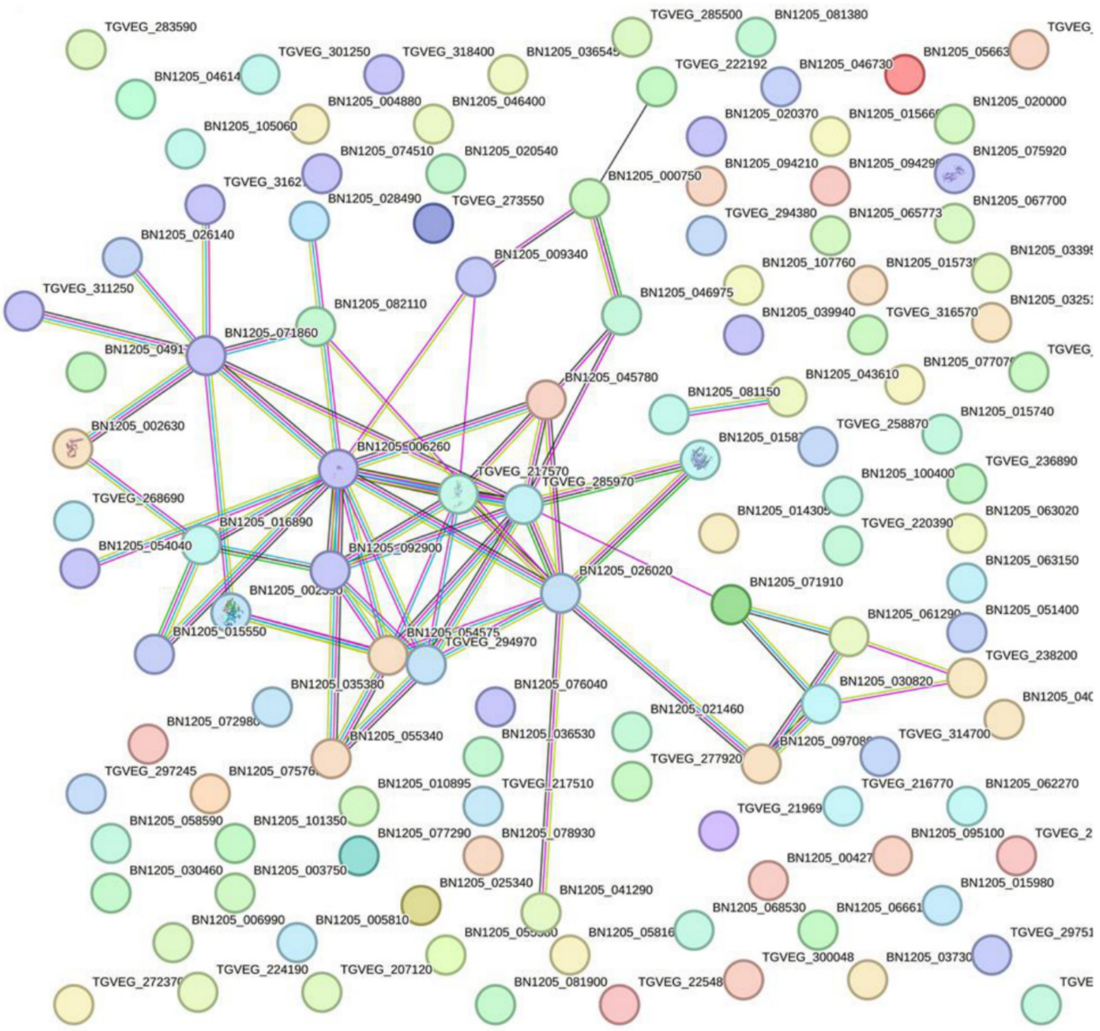

Supplementary Figure S5. Protein-protein interaction network of proteins differentially downregulated (59 proteins) in C3- and C4-clones compared to *T. gondii* wildtype tachyzoites. A unique network consisting of proteins involved in DNA replication and repair was revealed. The interaction network was created by the STRING knowledgebase and software tool from the Swiss Institute of Bioinformatics (www.expasy.org).

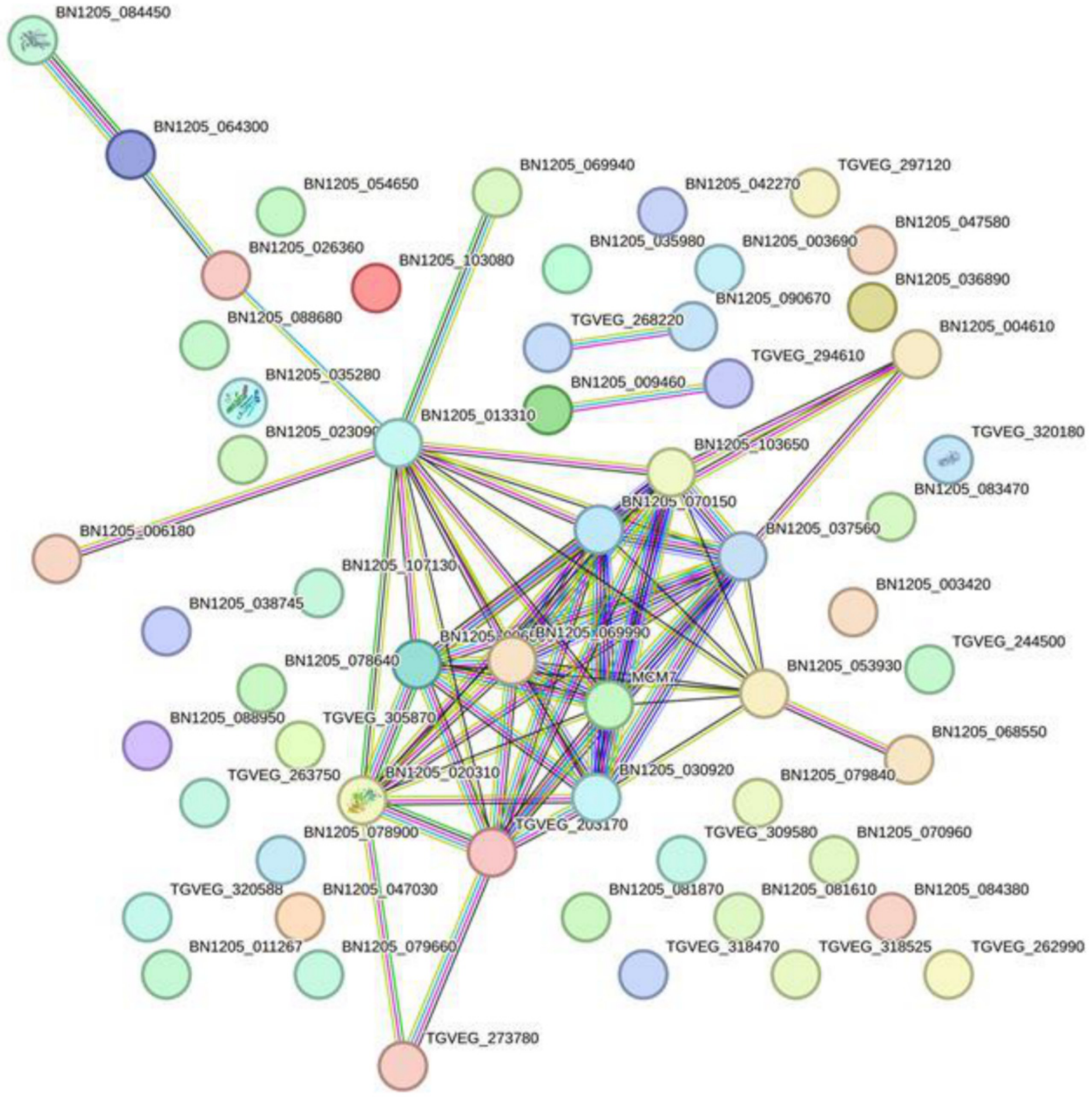

Supplement: Supplementary file 1 [file ijms-25-09067-s001.zip › ijms-3162973-supplementary.pdf]
